# Simulation of dry matter partitioning in cucumber fruits: reflecting gas exchange characteristics based on leaf position and cropping type

**DOI:** 10.1093/hr/uhaf124

**Published:** 2025-05-07

**Authors:** Ha Rang Shin, Yu Hyun Moon, Ha Seon Sim, Tae Yeon Lee, Soo Bin Jung, Yong Jun Kim, Na Kyoung Kim, JinWoo Lee, Tae Hyun Kim, Seunghyun Ban, Sung Kyeom Kim

**Affiliations:** Department of Horticultural Science, College of Agricultural and Science, Kyungpook National University, Daegu 41566, South Korea; Department of Horticultural Science, College of Agricultural and Science, Kyungpook National University, Daegu 41566, South Korea; Department of Horticultural Science, College of Agricultural and Science, Kyungpook National University, Daegu 41566, South Korea; Department of Horticultural Science, College of Agricultural and Science, Kyungpook National University, Daegu 41566, South Korea; Department of Horticultural Science, College of Agricultural and Science, Kyungpook National University, Daegu 41566, South Korea; Department of Horticultural Science, College of Agricultural and Science, Kyungpook National University, Daegu 41566, South Korea; Department of Horticultural Science, College of Agricultural and Science, Kyungpook National University, Daegu 41566, South Korea; Department of Horticultural Science, College of Agricultural and Science, Kyungpook National University, Daegu 41566, South Korea; Department of Horticultural Science, College of Agricultural and Science, Kyungpook National University, Daegu 41566, South Korea; Department of Horticultural Science, College of Agricultural and Science, Kyungpook National University, Daegu 41566, South Korea; Department of Horticultural Science, College of Agricultural and Science, Kyungpook National University, Daegu 41566, South Korea; World Horti Center, Kyungpook National University, Sangju 37224, Republic of Korea

## Abstract

This study aimed to predict dry matter partitioning in cucumber fruit (*Cucumis sativus* L.) by developing a simulation model that integrates photosynthetic characteristics based on leaf age and cropping type. Leaf gas exchange, growth, and environmental data from semi-forcing and forcing cropping types were used to calibrate models including the Farquhar-von Caemmerer-Berry (FvCB) model and other growth-related models. The FvCB model revealed reduced *V*_*cmax*_ and *J*_*max*_ values in older leaves across all cropping types, with semi-forcing crops showing higher photosynthetic capacities than forcing crops. Simulation results showed that, in predicting dry matter partitioning to fruit, the leaf-position-specific simulation model exhibited higher average *R*^2^ and lower RMSE (g m^−2^) compared to the leaf position-independent model, which applied the middle leaf FvCB model across all leaf ranks. Additionally, bias comparisons indicated greater consistency in the leaf-position-specific model. This approach allows growers to optimize environmental strategies by utilizing photosynthetic data form each leaf position. However, to further improve canopy-level predictions, future models should incorporate the temperature dependence of mesophyll conductance and the effects of photoperiodicity. This study underscores the value of integrating physiological and environmental complexities into crop simulation models, providing a foundation for enhanced predictions and the development of improved crop management strategies across various cultivation scenarios.

## Introduction

Crop simulation models enable the prediction of crop phenology and potential yields, helping optimize resource use, mitigate the effects of climate change, and analyze yield gaps [1]. In controlled environments, such as greenhouses, these models play a pivotal role in informing management decisions to optimize growing conditions and maximize both yield and economic returns [2]. Particularly, since fruit yield is directly dependent on the total amount of dry matter partitioned to the fruit, simulation models for dry matter partitioning to fruit are highly useful for predicting actual yield. In this context, dry matter partitioning does not merely indicate the relative proportion of dry matter allocated among organs but fundamentally represents the absolute allocation of assimilates produced through canopy photosynthesis. Since the total production of assimilates is directly governed by canopy photosynthesis, the absolute amount of dry matter partitioned to each organ is constrained by the total assimilate availability. Therefore, accurately simulating dry matter partitioning requires precise estimation of assimilate production, which is directly determined by the photosynthetic rate and capacity of the plant. By modelling this process, the simulation can provide a quantitative prediction of how much assimilate is actually allocated to each organ, particularly fruits, thereby enabling more reliable yield estimation.

A key element in dry matter partitioning simulation is the estimation of canopy photosynthesis. Canopy photosynthesis is a fundamental component of crop growth, influencing plant development, yield, and daily dry matter accumulation in crop models [3]. Typically, canopy photosynthesis is estimated by modelling photosynthetic responses at the single-leaf level, and then integrating and scaling these with leaf growth models, such as the leaf emergence and leaf area expansion models [4, 5].

The Farquhar-von Caemmerer-Berry (FvCB) photosynthesis model [6] is the most widely used model for predicting photosynthetic rates at the single-leaf level, allowing for the prediction of photosynthetic responses to changes in intercellular CO_2_ concentration (*C*_*i*_) [2, 7, 8]. However, accurately estimating photosynthetic rates across various canopy environments requires a more precise determination of intercellular CO_2_ concentration when using this model [2]. This accuracy can be enhanced by employing a coupled gas exchange model, which integrates the FvCB model with the Ball-Berry stomatal conductance model [9], representing the CO_2_ demand and supply functions, respectively [2, 10–17].

However, traditional models often generalize photosynthetic parameters and fail to account for critical factors such as leaf position, age, and environmental variations, which impact photosynthetic rates [15,18–25].

**Table 1:** Estimation results for *V*_*cmax25*_, *J*_*max25*,_ and *R*_*d25*_, along with model evaluation using calibration and validation data.

Cropping Type	Position	*V*_cmax25_	*J*_max25_	*R*_d25_	Calibration		Validation	
					*R* ^2^	RMSE	*R* ^2^	RMSE
Semi-forcing	Bottom	63.82	108.49	0.56	0.93	2.08	0.91	2.10
	Middle	74.35	126.40	0.35	0.96	1.80	0.92	2.27
	Upper	77.91	132.45	0.63	0.95	2.00	0.88	2.65
Forcing	Bottom	51.85	88.15	1.26	0.85	2.94	0.86	2.32
	Middle	67.06	114.00	1.36	0.91	2.58	0.90	2.32
	Upper	71.98	122.37	1.61	0.95	1.92	0.95	1.65

Previous research has shown that the decrease in nitrogen concentration in leaves with increasing age affects various leaf characteristics, including photosynthetic properties [2,26,27]. This reduction in nitrogen concentration depends on leaf position in crops with regular leaf axis patterns, such as cucumbers, indirectly suggesting that photosynthetic traits can serve as indicators of leaf age [22,27].

Additionally, studies have demonstrated that plants exhibit physiological adaptations and developmental changes based on temperature and light conditions, with photosynthesis also being influenced by these factors [28,29]. In greenhouse crops, like cucumber, transplanting times are adjusted to regulate production, resulting in cultivation under different cropping types.

Semi-forcing and forcing cropping systems are two distinct cultivation methods commonly used in greenhouse cucumber production to adjust harvest timing. Semi-forcing cropping involves relative earlier transplanting, allowing crops to grow under relatively moderate temperatures and longer photoperiods during the vegetative phase. In contrast, forcing cropping refers to much earlier transplanting under controlled environments with lower temperature and shorter photoperiods. These differences in cropping types clearly illustrate how plants at the same growth stage can be exposed to distinct environmental conditions, even with artificial environmental controls. Therefore, reflecting photosynthetic rates under different cropping types in simulation models can lead to more accurate predictions of canopy photosynthetic rates and yields. However, traditional canopy models focus on generalized parameters and do not adequately reflect the effects of leaf position and cropping systems under different growing conditions [30,31].

To address these gaps, this study aims to develop a canopy photosynthesis simulation model that incorporates leaf position and cropping type (semi-forcing and forcing types). This model provides more accurate predictions of canopy photosynthesis and dry matter partitioning compared to existing cucumber simulation models. It is anticipated that the findings of this study can be applied to various cultivation scenarios in the future, contributing to improved yield prediction and crop management strategies.

## Results

### Calibration and validation of FvCB models

To calibrate the FvCB model for each cropping type and leaf position, the model parameters, *V*_*cmax25*_, *J*_*max25*_, and *R*_*d25*_, were analyzed (Table 1). For all cropping types, both *V*_*cmax25*_ and *J*_*max25*_ decreased with increasing position rank, that is, with increasing leaf age. At all leaf positions, *V*_*cmax25*_ and *J*_*max25*_ were found to be higher for leaves grown in semi-forcing culture compared to those grown in forcing culture. The FvCB models were evaluated using the calibration data, and models for all cropping types and leaf positions demonstrated *R*^2^ values above 0.85 and RMSE values below 2.94 (μmol CO_2_ m^−2^ s^−1^) (Table 1; Fig. S1A in Supplementary Information 1). Subsequent validation with validation data demonstrated *R*^2^ values above 0.86 and RMSE values below 2.65 (μmol CO_2_ m^−2^ s^−1^) for all cropping types and leaf positions (Table 1; Fig. S1B in Supplementary information 1). Detailed evaluation results for each model using both calibration and validation data are provided in Table 1.

### Validation of coupled gas exchange models

The validation of the coupled gas exchange model for each crop type and leaf position revealed that, for photosynthesis rate (*A*), the *R*^2^ values for all crop types and leaf positions exceeded 0.78, with RMSE values below 3.29 (μmol CO_2_ m^−2^ s^−1^) (Table 2; Fig. S1C in Supplementary information 1). The prediction accuracy for *C*_*i*_ showed an *R*^2^ value of 0.93 and an RMSE below 64.33 (μmol mol^−1^) for all cropping types and leaf positions (Table 2; Fig. S1D in supplementary information 1). Detailed evaluation results of the coupled gas exchange model in predicting *A* and *C_i_* values are presented in Table 2.

**Table 2:** Validation results of the coupled gas exchange model for photosynthesis rate (*A*) and intercellular CO_2_ concentration (*C_i_*) based on cultivation type and leaf position

Validation variable	Cultivation type	Position	*R* ^2^	RMSE
*A*	Semi-forcing	Bottom	0.78	3.29
		Middle	0.85	3.06
		Upper	0.83	3.16
	Forcing	Bottom	0.85	2.45
		Middle	0.87	2.64
		Upper	0.91	2.07
*C_i_*	Semi-forcing	Bottom	0.93	64.3
		Middle	0.97	41.9
		Upper	0.97	46.6
	Forcing	Bottom	0.96	49.7
		Middle	0.97	42.4
		Upper	0.99	32.9

### Results of the node and leaf emergence model and the leaf expansion model

The model for predicting node and leaf emergence was divided into two phases: the vegetative-only phase during which all flowers were removed, and the phase where both vegetative and reproductive growth occurred simultaneously, which began after the flowering of the 10th phytomer. The apex thermal time threshold (AT_threshold_) to produce a new phytomer was modelled as 26.3°Cd during the vegetative-only phase, and 15.6°Cd during the phase of simultaneous vegetative and reproductive growth.

**Figure 1:**
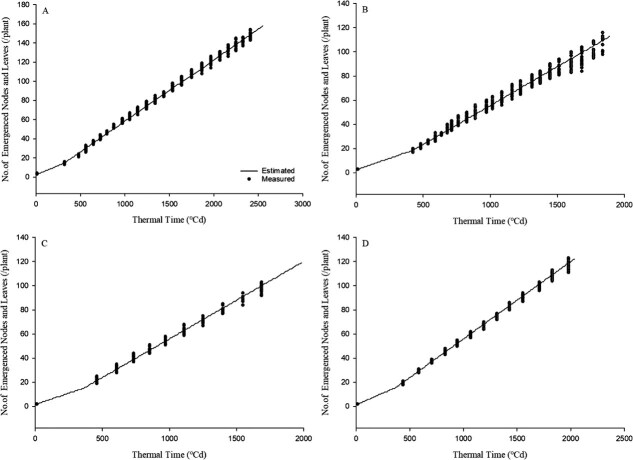
Estimated and observed number of nodes and leaves emerged by thermal time (°Cd) as estimated by the mode and leaf emergence model. Thermal time (°Cd). A, B, C, and D are the results and data from experiments 1, 2, 3, and 4, respectively. The detailed model performance metrics (*R*^2^ and RMSE) for each experiment are described in the Results section.

When evaluated against the cumulative node and leaf data from experiment 1, the model achieved an *R*^2^ value of 0.99 and an RMSE value of 2.48. For experiments 2, 3, and 4, the *R*^2^ values were 0.98, 0.99, and 0.99, respectively, with corresponding RMSE values of 3.78, 2.56, and 2.12 (nodes per plant) (Fig. 1).

**Figure 2:**
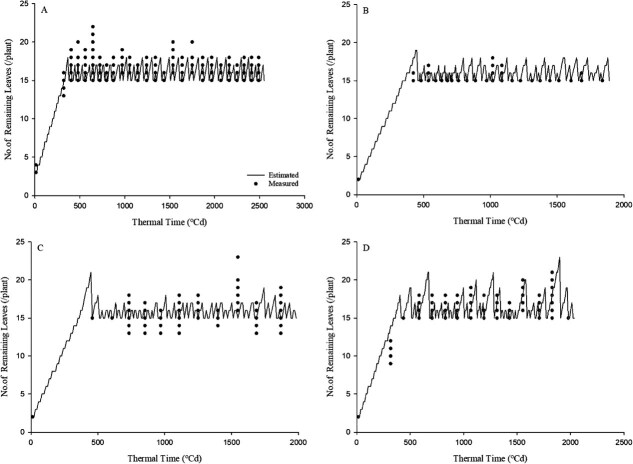
Estimated and observed number of remaining leaves by thermal time (°Cd) as estimated by the node and leaf emergence model with defoliation code. Thermal time (°Cd). A, B, C, and D are the results and data from experiments 1, 2, 3, and 4, respectively. The detailed model performance metrics (*R*^2^ and RMSE) for each experiment are described in the Results section.

The simulation evaluation of the actual number of leaves remaining on the plant, reflecting the defoliation process during the experiments, yielded *R*^2^ values of 0.49, 0.78, 0.79, and 0.60 for experiments 1, 2, 3, and 4, respectively, with RMSE values of 1.13, 0.69, 1.52, and 1.77 (leaves per plant), respectively (Fig. 2).

**Figure 3:**
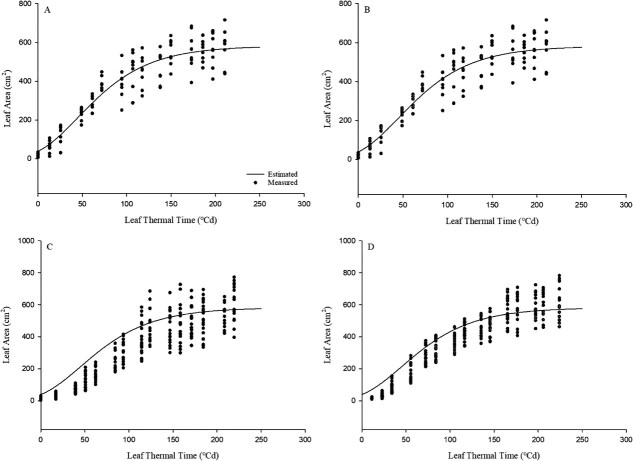
Estimation results of leaf area and measured data using leaf expansion model by leaf thermal time (°Cd). A, B, C, and D are the results and data from experiments 1, 2, 3, and 4, respectively. The detailed model performance metrics (*R*^2^ and RMSE) for each experiment are described in the Results section.

The leaf area expansion model, based on the thermal time of individual leaves, was developed using data from experiment 1. The parameters a, b, and c in Equation (14) were estimated to be 582.06, 45.33, and 45.31, respectively. The model was evaluated against the modelled data, resulting in an *R*^2^ value of 0.87 and an RMSE of 69.40 (cm^2^). When applied to experiments 2, 3, and 4, the *R*^2^ values were 0.77, 0.71, and 0.85, respectively, with RMSE values of 86.37, 113.34, and 83.93 (cm^2^), respectively, indicating predictive accuracy (Fig. 3).

### Simulation results of dry matter partitioning to fruit

**Figure 4:**
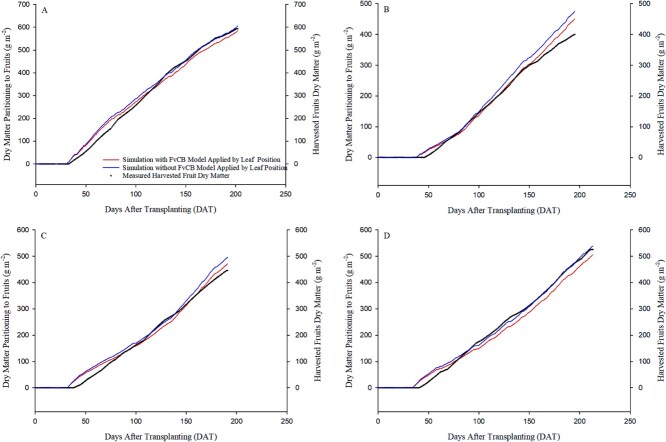
Simulation results of cumulative dry matter partitioned to fruit and measured cumulative harvested fruit dry weight by days after transplanting (DAT). A, B, C, and D represent the results from experiments 1, 2, 3, and 4, respectively. The left *y*-axis represents the simulated cumulative dry matter partitioned to fruit (g m^−2^), while the right *y*-axis represents the measured cumulative harvested fruit dry weight (g m^−2^). The line labeled “Simulation with FvCB Model Applied by Leaf Position” and “Simulation without FvCB model Applied by Leaf Position” represent the leaf- position-specific and leaf-position-independent models, respectively. Dots represent the measured cumulative harvested fruit dry weight (g m^−2^), which was initially collected as daily data. The detailed model performance metrics (*R*^2^ and RMSE) are described in the Results section.

The predicted initial dry weight for experiments 1, 2, 3, and 4 were 0.67, 0.29, 0.13, and 0.22 g per plant, respectively. Using the leaf-position-specific model, the daily partitioned dry matter to fruit was accumulated to estimate the cumulative dry matter partitioned to fruit. The estimated values were then compared to the measured cumulative dry weight of harvested fruit. As a result, the *R*^2^ values were 0.99 for experiments 1, 2, and 3 and 0.98 for experiment 4. The RMSE for each experiments were 19.02, 14.60, 15.11, and 21.56 (g m^−2^), respectively (Fig. 4).

In contrast, the leaf-position-independent model, which applies the middle leaf FvCB model uniformly across all leaf positions, yielded *R*^2^ values of 0.99, 0.96, 0.98, and 0.99 and RMSE values of 21.17, 26.54, 22.17, and 12.45 (g m^−2^) for experiments 1, 2, 3, and 4, respectively (Fig. 4).

To quantitatively analyze the predicted dry matter partitioning for the entire plant, further evaluations were performed using leaf-position-specific and leaf-position-independent models. Model performance was evaluated by comparing the total dry matter partitioned to fruit during the experiment predicted by each model to the cumulative dry weight of harvested fruit measured at the end of the experiments (Table 3). In addition, to evaluate the performance of the simulated vegetative dry matter predictions, total vegetative dry matter predicted by both models at the experiment endpoint was compared to the measured vegetative dry weight obtained through destructive sampling (Table 4).

Since root dry weight was not directly measured at the end of the experiment, it was estimated using a linear interpolation method based on the temperature-dependent root to total vegetative dry weight ratio reported in previous study [32] [Supplementary Information 2]. This estimate was incorporated into the measured vegetative dry weight to improve the accuracy of the model evaluation.

In the evaluation of final cumulative fruit dry matter partitioning prediction evaluation, the predictions (Table 3). Compared to the leaf-position-independent model, it had lower RMSE (g m^−2^) and MAE (g m^−2^) values in most experiments, demonstrating higher prediction accuracy and confidence (Table 3). In contrast, the leaf-position-independent model tended to overestimate, which contributed to higher RMSE and MAE than the leaf-position-specific model (Table 3).

In the evaluation of vegetative (leaf, stem, petioles, and root) dry matter prediction, the leaf-position-specific model consistently underestimated in all experiments, with negative bias values, and had higher RMSE and MAE than the leaf-position-independent model (Table 4). In contrast, the leaf-position-independent model exhibited a mixed bias trend, with alternating overestimation and underestimation depending on the experiment (Table 4). Overall, while RMSE and MAE were lower than those of the leaf position model, its predictive stability was lower, exhibiting greater variability across experiments (Table 4).

## Discussion

### FvCB models based on leaf age and cropping type

Previous studies have demonstrated that shaded leaves in the lower canopy of various crops exhibit reduced Rubisco content and diminished RuBP regeneration capacity [27,33,34]. Furthermore, as leaves age, nitrogen content decreases, resistance to CO_2_ diffusion increases due to the accumulation of nonstructural carbohydrates, and light absorption decreases because of changes in chloroplast structure. These factors contribute to a reduction in Rubisco activity [26]. Consistent with these earlier findings, this study also observed a tendency for both the maximum carboxylation capacity and the maximum electron transport rate to decrease with leaf age across all cropping types.

In this study, the *V*_*cmax*_ and *J*_*max*_ values of cucumber leaves in the semi-forcing cropping type experiment were found to be higher than those of cucumber leaves in the forcing cropping type experiment. The average temperature throughout the growing period of experiment 1, which involved a semi-forcing cropping type in which photosynthesis measurement experiments were conducted, was higher than that of experiment 2, which utilized a forcing cropping type. Specifically, the optimum growing temperature ranges were 24.70 ± 1.78°C for the semi-forcing cultivation type and 23.65 ± 2.0°C for the forcing cultivation type. Within the growth temperature range, crops adapt and adjust to optimal temperatures that vary depending on the conditions in which they are grown [35]. Additionally, electron transport capacity increases due to enhanced membrane fluidity, elevated ATP synthase activity, and increased molecular movement at relatively high temperatures [35]. Thus, it can be inferred that *V*_cmax_ and *J*_max_ were higher in the semi-forcing type grown at relatively high temperatures across all leaf positions, despite photosynthesis being measured under the same conditions in both cropping type experiments.

### Leaf emergence and growth

According to previous studies, the thermal time required for the cucumber apex to generate a new phytomer, including both the node and leaf, has consistently been modelled at 16.7°Cd, regardless of the growth stage [36]. However, calibrating this model with data from our study revealed a notable difference in the thermal time required before and after the onset of reproductive growth. Specifically, the thermal time was 26.3°Cd prior to reproductive growth and decreased to 15.6°Cd afterwards. The variation in the required thermal time of node emergence across growth stages can be attributed to the allocation of resources during the early vegetative stage. During this phase, relatively more resources are directed towards root development to facilitate adaptation to the environment, which may subsequently slow down node development compared to the period following the onset of reproductive growth [37].

Furthermore, as the plant transitions to reproductive growth, physiological changes, such as hormonal shifts, can accelerate the rate of node development. For example, the onset of reproductive growth may have increased the concentration of gibberellin, which is important for the development of flowers and reproductive organs [38]. This may have induced the degradation of DELLA proteins and activation of the SQUAMOSA PROMOTER BINDING PROTEIN-LIKE (SPL) transcription factor, which promoted the development of nodes and stems in the apical meristem and accelerated node formation during reproductive growth [38].

This study employed a Gompertz growth curve, based on thermal time, to model leaf area expansion. The Gompertz growth curve is widely utilized to illustrate growth patterns across a variety of organisms [39]. A previous study involving tomato, an indeterminate growth crop similar to cucumber, confirmed that this model, using growth degree days (GDD) as a variable, effectively describes leaf area growth over time [40].

Moreover, the SLA (m^2^ g^−1^) for the semi-forcing cropping type was measured at 0.025 (±0.0005 standard error) compared to 0.018 (± 0.0004 standard error) for the forcing cropping type, indicating a significant difference between the two cropping systems. In general, *V*_cmax_ and *J*_max_ are proportional to nitrogen concentration and inversely related to SLA [41]. However, in this study, the semi-forcing cropping type exhibited an anomaly wherein *V*_cmax_ and *J*_max_ values were higher than those observed in the forcing cropping type, despite a greater SLA. This result is likely attributable to the fact that SLA is influenced by various environmental factors, including temperature, light, and nutrient availability, in addition to photosynthetic efficiency [42,43]. According to a study by Wang *et al.*, the SLA of cucumber seedlings decreased by 62.8% as daily light integral increased from 8.64 to 28.80 mol^−2^ d^−1^ [44]. Other research showed that cucumber with the highest fruit load had an SLA of 0.0179 m^2^ g^−1^, which decreased to 0.0131 m^2^g^−1^ when all fruit was removed [45]. This decrease in SLA was associated with decreased dry weight of fruit. In particular, the semi-forcing cultivation system, which is characterized by rapid growth rates, likely had higher SLA because it requires greater sink (sink, an organ that consumes or stores photosynthetic products) demand for rapid biomass accumulation. The increased SLA suggests that, despite favorable light conditions, thinner leaves were maintained to support accelerated growth and higher assimilate demand. Notably, since we collected leaf area and leaf dry weight data solely from destructive surveys conducted at the end of the experiments; it is possible that the SLA measured at that time was not representative of the entire growing period. This measurement may only reflect the environmental conditions and growth characteristics of the remaining leaves at that specific time. Therefore, future studies should consider the effects of varying environmental conditions on leaf development and SLA to gain a more comprehensive understanding of these results.

### Position-specific photosynthesis models for canopy simulation: Pathway to more accurate prediction

The simulation results of cumulative dry matter partitioned to fruit throughout the experimental period, as predicted by the leaf-position-specific and leaf-position-independent models, were compared with the measured cumulative harvested fruit dry weight (Fig. 4). The leaf-position-specific model showed an average *R*^2^ value of 0.99 for experiments 1, 2, 3, and 4, while the leaf-position-independent model showed an average *R*^2^ value of 0.98. Furthermore, the average RMSE was 17.57 g m^−2^ for the leaf-position-specific model and 20.58 g m^−2^ for the leaf-position-independent model, indicating that the leaf-position-specific model was more accurate in predicting dry matter partitioning to fruit (Fig. 4). These results suggest that a model incorporating the photosynthetic characteristics of each leaf can predict the partitioning of dry matter to fruit more precisely than a model based on a single representative leaf (leaf-position-independent model).

The final cumulative dry matter partitioned to fruit predicted by each model at the experiment endpoint was compared with the total harvested fruit dry weight (Table 4). The leaf-position-specific model showed values closer to the actual measurements, while the leaf-position-independent model showed both underestimation and over estimation trends across experiments, suggesting a lack of consistency in predicting dry matter partitioning to fruit (Table 4).

**Table 3:** Evaluation of model performance through a comparison of measured cumulative harvested fruit dry weight at the experiment endpoint and the simulated final cumulative dry matter partitioned to fruit.

EXP	Model	Prediction (g m^−2^)	RMSE (g m^−2^)	MAE (g m^−2^)	Bias (g m^−2^)
1	Leaf position specific	582.46	51.18	44.99	−10.01
	Leaf position independent	602.27	51.15	45.45	7.88
	Measured data (Mean ± SD)	592.47 ± 51.28	–	–	–
2	Leaf position specific	448.59	64.24	53.49	53.49
	Leaf position independent	472.93	85.58	77.83	77.83
	Measured data (Mean ± SD)	395.09 ± 36.73	–	–	–
3	Leaf position specific	471.08	53.15	44.41	−4.21
	Leaf position independent	496.22	56.97	44.41	20.93
	Measured data (Mean ± SD)	475.29 ± 55.34	–	–	–
4	Leaf position specific	505.57	51.63	40.93	−22.95
	Leaf position independent	539.32	47.50	38.22	10.80
	Measured data (Mean ± SD)	528.52 ± 47.45	–	–	–

The leaf-position-specific model and the leaf-position-independent model were evaluated based on RMSE, MAE, and Bias.

**Table 4:** Evaluation of model performance through a comparison of measured total vegetative dry weight (leaves, stem, petioles, and root) at the experiment endpoint and the simulated total vegetative dry matter at the experiment endpoint.

EXP	Model	Prediction (g m^−2^)	RMSE (g m^−2^)	MAE (g m^−2^)	Bias (g m^−2^)
1	Leaf position specific	275.31	48.63	46.59	−31.29
	Leaf position independent	299.85	37.83	27.50	−6.75
	Measured data (Mean ± SD)	306.60 ± 39.48	–	–	–
2	Leaf position specific	196.62	26.86	22.60	−20.80
	Leaf position independent	232.37	22.64	18.81	14.96
	Measured data (Mean ± SD)	217.41 ± 17.60	–	–	–
3	Leaf position specific	161.51	96.95	90.93	−90.93
	Leaf position independent	193.35	56.97	44.41	−58.79
	Measured data (Mean ± SD)	252.14 ± 34.73	–	–	–
4	Leaf position specific	197.01	46.21	41.78	−41.78
	Leaf position independent	242.88	20.17	16.92	4.09
	Measured data (Mean ± SD)	238.79 ± 20.26	–	–	–

The leaf-position-specific model and the leaf-position-independent models were evaluated based on RMSE, MAE, and Bias.

**Table 5:** Overview of the experimental period, cultivar, and cropping type

Exp.	Cultivar		Transplant date (yyyy mm dd)	End date (yyyy mm dd)	Cropping Type
	Scion	Rootstock			
1	*‘Goodmoringbaekdadagi’* ^a^	*‘Heukjong’* ^b^	23 February 2021	13 September 2021	Semi-forcing
2	*‘Hangangmatbaekdadagi’* ^c^	*‘Heukjong’*	19 October 19	2 May 2022	Forcing
3	*‘Hangangmatbaekdadagi’*	*‘Heukjong’*	8 October 2022	17 April 2023	Forcing
4	*‘Hangangmatbaekdadagi’*	*‘Heukjong’*	14 October 2023	4 May 2024	Forcing

a Cucumber (*Cucumis sativus* L., Nongwoobio Co., Ltd., Korea)

b Squash (*Cucurbita ficifolia Bouche*., ASIA SEED Co., Ltd., Korea)

c Cucumber (*Cucumis sativus* L., FarmHannong Co., Ltd., Korea)

Similarly, when comparing predictions of vegetative dry matter and measurements of vegetative dry weight, the leaf-position-specific model showed negative bias values and consistently underestimated (Table 5). In contrast, the leaf-position-independent model showed a mixed bias trend and generally had relatively better predictive performance with lower RMSE and MAE. However, since this evaluation is based on a single time point rather than cumulative values, it mainly evaluates the prediction accuracy at the end of the experiment rather than the total assimilation production of the entire simulation.

Therefore, considering these results comprehensively, the leaf-position-specific model is more suitable for predicting dry matter partitioning to fruit. However, future studies should integrate both total dry matter production and its partitioning to vegetative organs to more comprehensively evaluate overall model performance.

In addition, utilizing the leaf-position-specific model enables growers to leverage photosynthesis data by position on each leaf to optimize strategies for control temperature, leaf pruning, CO₂ dosing and control screen, thereby improving light utilization efficiency and distribution of products made from photosynthesis. This approach not only increases crop yield and resource efficiency in a controlled environment but also provides a scientific foundation for sustainable horticultural production.

### Addressing model limitations and future directions

Most dry matter partitioning simulations do not consider mesophyll conductance (*g_m_*). This is because the parameters of the photosynthesis model are geared to the *C_i_*, not to CO_2_ concentration inside the chloroplast [46,47]. However, recent advances in gm measurement technology have shown that gm can significantly limit the rate of photosynthesis (*A*), and its effects vary with temperature. Significant differences have been reported in these reactions [46,48]. In particular, von Caemmerer, Niinemets *et al.*, Sun *et al.*, and Rogers *et al.* reported that *V*_cmax_ would be underestimated if *g_m_* was not taken into account in the *A*-*Ci* curve analysis [28,46,49,50]. Previous studies have shown that when leaf temperature is 25°C, considering g_m_ increases *V*_cmax_ by 23.4% [47]. The widely used theory of gas exchange by von Caemmerer and Farquhar is a useful model, but it has some limitations [51]. This model effectively integrates mole fluxes, mole fraction gradients, and outer effects, but does not consider important aspects such as cuticular fluxes, the heterogeneity of leaf surface conditions, and the ternary effects within the boundary layer. This omission may lead to an error in the measurement of *C_i_*, which is an important parameter in photosynthesis studies [52]. Bauerle *et al.* observed that day and night lengths are more reliable indicators than temperatures in predicting seasonal changes in *V*_cmax_ and *J*_max_ [22]. Specifically, *V*_cmax_ peaks immediately after the summer solstice, and gradually decreases as it enters autumn, even if the temperature continues to rise for more than a month. This suggests that the photoperiod should not be overlooked when determining photosynthetic parameters. In addition, as mentioned in the ‘FvCB models based on leaf age and cropping type’, the photosynthetic optimum temperature may change due to long-term growth temperature adaptation [53]. The reason that dry weight predictions were underestimated or overestimated in the simulation results of this study is likely due to the overlooked *g_m_*, insufficient consideration of the photoperiod effect, insufficient reflection of the long-term temperature adaptation, and limitations of the gas exchange model currently used. To improve the future model, it is essential to include a temperature dependence of *g_m_*, consider the effect of the photoperiod on seasonal changes, reflect long-term temperature acclimation, and more accurately reflect the complexity of the photosynthesis process within the gas exchange model.

Additionally, MAE, an evaluation metric that has the advantage of being less affected by outliers when applying simulation models, and quickly identifying differences in predictions that may be caused by environmental changes or crop characteristics, can be used to assess how well a model is tracking actual growth as environmental factors such as air temperature, light, and carbon dioxide (CO_2_) concentration change, or as regional and crop-specific conditions change.

Additionally, when MAE, an evaluation metric that is less affected by outliers and has the advantage of quickly identifying prediction differences caused by environmental changes or crop characteristics, is applied together with the simulation model, it can be used to assess how well the model tracks actual growth. Furthermore, it can be utilized to improve the model as environmental factors such as temperature, light, and CO₂ concentration change, or as regional and crop-specific conditions vary.

To include these improvements, more fine parameter settings and flexible structures that can reflect the specific characteristics of a crop or system are needed. By incorporating advanced methods for *g_m_* measurements, adjusting them to long-term temperature changes, and reflecting seasonal changes that vary with day length, future models can more accurately predict crop yields, ultimately informing better resource management and strategic decision-making in both research and commercial agricultural systems.

## Conclusion

This study developed and validated a simulation model of canopy photosynthesis that incorporated leaf position and cropping system-specific parameters to significantly improve dry matter partitioning predictions in cucumber fruit. The results revealed physiological differences characterized by higher *V*_cmax_ and *J*_max_ values under semi-forcing conditions. From these observations, it could be inferred that such differences are linked to adaptation to optimal temperatures. Leaf-position-specific modelling showed higher accuracy compared to traditional generalized models, providing a robust framework to improve crop yield prediction.

By addressing the limitations of existing canopy models, this study highlights the importance of incorporating leaf position and cropping type-specific characteristics into crop simulations. This approach not only advances the understanding of crop physiology in a controlled environment, however, also provides actionable insights to optimize resource use and improve productivity.

However, this study did not consider factors such as mesophyll conductance, photoperiod effects, and long-term temperature acclimation, which can influence photosynthetic performance under various conditions. Incorporating these variables in future research could further enhance the model’s predictive accuracy. Additionally, extending the model to other crops with similar physiological traits could broaden its applicability in crop simulation studies.

This study not only advances our understanding of cucumber physiology under controlled conditions but also serves as a template for developing crop simulation models that integrate physiological and environmental complexities (Table S1 in Supplementary information 3). As agricultural systems face increasing pressures from climate variability, such refined models will play a critical role in ensuring sustainable and productive crop management strategies.

## Materials and methods

### Description of models

#### Coupled gas exchange model

To simulate leaf-level gas exchange coupled gas exchange model that integrates Farquhar-von Caemmerer-Berry (FvCB) photosynthesis model and Ball-Berry stomatal conductance model.

#### FvCB photosynthesis model

In the FvCB model, the net photosynthesis rate (*A*, μmol CO_2_ m^−2^ s^−1^) is predicted based on the assumption that it is limited by the slower of two biochemical processes:


(1) \begin{equation*} A=\mathit{\min}\left\{{A}_c,{A}_j\right\} \end{equation*}

*A_c_* is the net photosynthesis rate when limited by Rubisco carboxylation (μmol m^−2^ s^−1^), and *A*_*j*_ is the net photosynthesis rate when ribulose-1,5-bisphosphate (RUBP)-regeneration is limited by electron transport (μmol m^−2^ s^−1^). The Rubisco-limited photosynthesis rate, *A*_*c*_, is given by:


(2) \begin{equation*} {A}_c=\frac{V_c\times \left({C}_i-{\varGamma}^{\ast}\right)}{C_i+{K}_c\left(1+\frac{O}{K_o}\right)}-{R}_d \end{equation*}

where *V_c_* (μmol m^−2^ s^−1^) is the carboxylation capacity under a given light intensity (μmol m^−2^ s^−1^), *C_i_* (μmol mol^−1^) and *O* (210 mmol mol^−1^) are the intercellular concentrations of CO_2_ and O_2_, respectively, *K_c_* (μmol mol^−1^), and *K_o_* (mmol mol^−1^) are the Michaelis–Menten constants for Rubisco activity for CO_2_ and O_2_, respectively, ${\varGamma}^{\ast }$ (μmol mol^−1^) is the CO_2_ compensation point in the absence of mitochondrial respiration, and *R_d_* (μmol CO_2_ m^−2^ s^−1^) is the rate of day respiration (nonphotorespiratory). The electron transport-limited photosynthesis rate, *A_j_*, is expressed as:


(3) \begin{equation*} {A}_j=\left(\frac{J\times \left({C}_i-{\varGamma}^{\ast}\right)}{4{C}_i+8{\varGamma}^{\ast }}\right)-{R}_d \end{equation*}

(4) \begin{equation*} J=\frac{\alpha \times \mathrm{PPFD}+{J}_{\mathrm{max}}-\sqrt{{\left(\alpha \times \mathrm{PPFD}+{J}_{\mathrm{max}}\right)}^2-4\mathrm{\theta} \times{J}_{\mathrm{max}}\times \mathrm{\alpha} \times \mathrm{PPFD}}}{2\theta } \end{equation*}

where *J* is the rate of electron transport rate (μmol m^−2^ s^−1^), *J*_max_ is the maximum electron transport rate (μmol m^−2^ s^−1^), α is the efficiency of light energy conversion on an incident light basis, and θ is the curvature of the light response curve of *J* (dimensionless).

The parameter *V_c_* is equivalent to *V*_*c*max_ (mol m^−2^ s^−1^), representing the maximum carboxylation capacity when Rubisco is fully activated. The activation of Rubisco as a function of light intensity is reflected using the expression in Equation (S4-1) in Supplementary Information 4, and the estimated activation response based on our data is shown in Fig. S4 in Supplementary Information 4.

The temperature response function [54] for parameters *K_c_*, *K_o_*, and ${\varGamma}^{\ast }$ is represented by Equation (5):


(5) \begin{equation*} \mathrm{Parameter}={\mathrm{Parameter}}_{25}\ \exp \left[\frac{H_a\left({T}_k-298.15\right)}{\left(298.15\times R\times{T}_k\right)}\right] \end{equation*}

where *T_k_* is the leaf temperature in K, *H*_*a*_ (J mol^−1^) is the activation energy, and *R* is the universal gas constant (8.314 mol^−1^ K^−1^).

The temperature response for parameters *V*_*c*max_ and *J*_max_ is represented by a peaked function, which is a modified version of the Arrhenius function, as shown in Equation (6):


(6) \begin{align*}\begin{aligned} &\mathrm{Parameter}\\&={\mathrm{Parameter}}_{25}\ \exp \left[\frac{H_a\left({T}_k-298.15\right)}{\left(298.15\times R\times{T}_k\right)}\right]\ \frac{1+\exp \left(\frac{T_k\times S-{H}_d}{298.15\times R}\right)}{1+\exp \left(\frac{T_k\times S-{H}_d}{T_k\times R}\right)} \end{aligned}\end{align*}

where *H_d_* (J mol^−1^) represents the deactivation energy, and *S* (J K^−1^ mol^−1^) is the entropy factor.

The temperature response for the parameter *R_d_* is represented by Equation (7), which is based on the *Q*-10 model [55]:


(7) \begin{equation*} {R}_d={Q_{10-\mathrm{value}}}^{\left(\frac{T-25}{10}\right)}\times{R}_{d25} \end{equation*}

where *T* is the leaf temperature in degrees Celsius, *R*_*d*25_ is the respiration rate at 25°C, and the *Q*_10-value_ is 2. All parameters, symbols, and values of the FvCB model are detailed in Table A1. Additionally, the temperature response parameters for *K_c_*, *K_o_*, ${\varGamma}^{\ast }$, *V*_*c*max_, and *J*_max_ are listed in Table A2.

#### Stomatal conductance model

The stomatal conductance is estimated using Equation (8) which is based on the Ball-Berry model [9]:


(8) \begin{equation*} {g}_{\mathrm{sw}}=m\frac{h}{C_s}\mathrm{A}+{g}_{\mathrm{sw}\mathrm{min}} \end{equation*}

where *g*_sw_ is the stomatal conductance of H_2_O (mol m^−2^ s^−1^), *g*_swmin_ is the minimum stomatal conductance of H_2_O (mol m^−2^ s^−1^), h is the relative humidity at the leaf surface (unitless), m is the slope parameter, and *c_s_* is the leaf surface CO_2_ concentration.

According to Ball [56], if the effect of water efflux from leaves on the diffusion of CO_2_ into leaves is neglected, the value of *C_i_* can be calculated using Equation (9):


(9) \begin{equation*} {C}_i={C}_s-\frac{A}{g_{sc}} \end{equation*}

where *g*_sc_ is the stomatal conductance of CO_2_. All parameters, symbols, and values used in the Ball-Berry model are presented in Table B1.

#### Coupling of photosynthesis and stomatal conductance models

**Figure 5:**
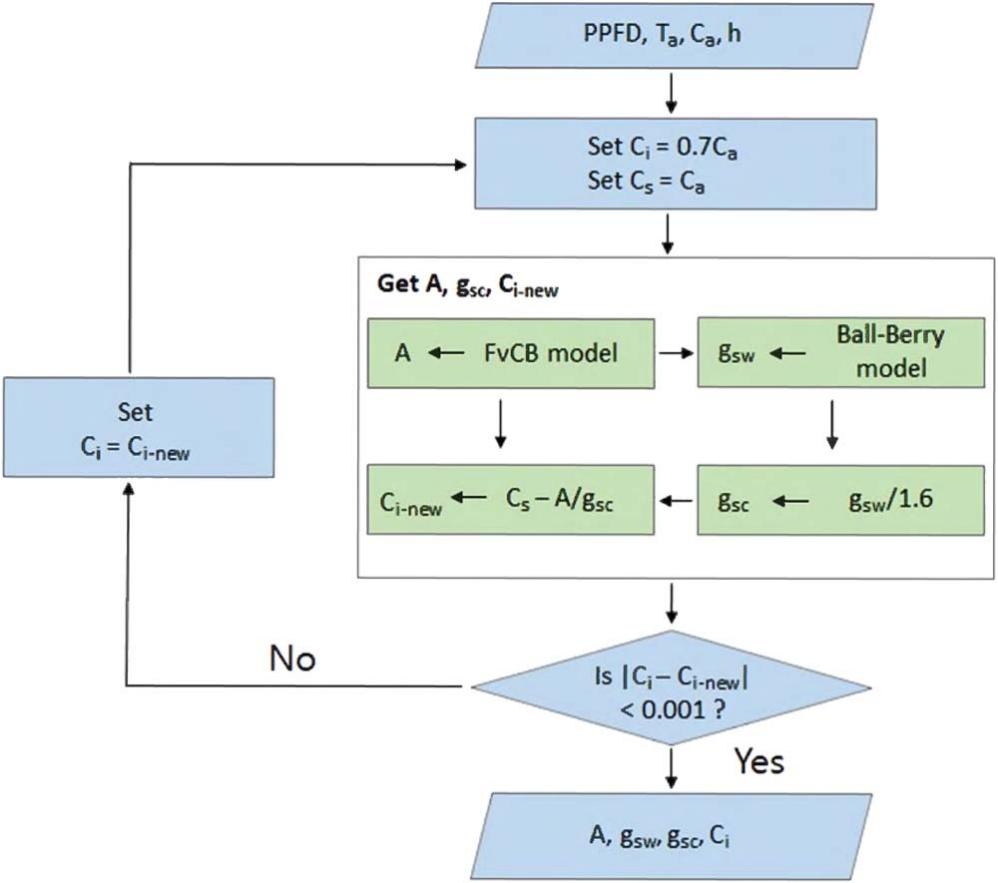
Schematic diagram of the coupled gas exchange model flow.

The FvCB model and the Ball-Berry model were used as submodels to form a coupled gas exchange model [2, 17]. These two submodels are interdependent, and a nested iterative approach was employed to address the relationships among the submodel equations. Initially, the value of *C_i_* was estimated at 70% of *C_a_*, which was integrated into the FvCB model to initiate the calculation (Equation (1)) and obtain an estimated value for A [2, 17]. Next, assuming *C_s_* to be equal to *C_a_* (the concentration of CO_2_ in air), the stomatal conductance for H_2_O (*g*_*sw*_), was calculated using the Ball-Berry model (Equation (8)). A conversion factor of 1.6, derived from the energy equation (Equation (10)), was applied to derive the stomatal conductance for CO_2_ from the stomatal conductance for H_2_O (*g*_sc_) [17]. Using the estimated *A*, *g*_sc_, and another stomatal conductance equation (Equation (9)), a new value for *C_i_* was calculated. This calculation proceeded through iterative steps, applying the Newton–Raphson technique until the variation in *C_i_* fell below a tolerance threshold of 0.001 [2, 17]. A schematic of the coupled gas exchange model is shown in Fig. 5.


(10) \begin{equation*} {g}_{sc}=\frac{g_{sw}}{1.6} \end{equation*}

#### Node and leaf emergence model and leaf expansion model

Accurate simulation of canopy photosynthesis requires precise estimation of individual leaf area, as it is a key determinant of light interception and total canopy photosynthetic capacity. To incorporate this factor into the model, a node and leaf emergence model was developed to determine the timing of new leaf formation, while a leaf area expansion model was designed to simulate the dynamic growth of individual leaves over time. By integrating these models, rank-specific leaf area was dynamically estimated and incorporated into the simulation framework for canopy photosynthesis and dry matter partitioning.

Both models adopt a thermal time-based approach, assuming a base temperature of 10°C [36]. The thermal time equation is presented in Equation (11):


(11) \begin{equation*} TT\ \left({}^{{}^{\circ}} Cd\right)=\sum \max \left({T}_{\mathrm{avg}}-{T}_{\mathrm{base}},0\right) \end{equation*}

where *TT* represents thermal time (°Cd), *T*_avg_ is daily average temperature (°C), and *T*_base_ is base temperature.

#### Node and leaf emergence model

In the node and leaf emergence model, apex thermal time (*AT*) regulates the initiation of new phytomers. The apex continuously develops, and once accumulated apex thermal time reaches a specific threshold (*AT*_threshold_), the apex generates a new phytomer, which includes a leaf and a node. This process is expressed by Equation (12):


(12) \begin{equation*} A\left( AT, nn\right): AT\ge{AT}_{threshold}\to \left\{A\left(0, nn+1\right),N\left( nn+1\right),L\left( nn+1\right)\right\} \end{equation*}

where $A\left(0, nn+1\right)$ represents the newly formed apex with a thermal time reset to 0, while $N\left( nn+1\right)$ and $L\left( nn+1\right)$ denote the newly generated node and leaf, all assigned the phytomer rank $nn+1$.

#### Leaf expansion model

Following emergence, each leaf accumulates its own thermal time ($LT$) independently, starting from zero at its appearance. The leaf area expansion model describes the growth of individual leaf area as a function of $LT$ using a Gompertz growth curve (Equation (13)) [36]:


(13) \begin{equation*} \mathrm{Leaf}\ \mathrm{area}\ \left({cm}^2\right)=a\times \exp \left(-\mathit{\exp}\left(-\frac{\left(- LT-b\right)}{c}\right)\right) \end{equation*}

The parameter $a$ defines the maximum potential leaf area, while $b$ and $c$ are empirical parameters. By tracking each leaf independently, the model enables a rank-specific estimation of leaf area.

#### Simulation of canopy photosynthesis and dry matter partitioning to fruit

To simulate canopy photosynthesis and dry matter partitioning, a coupled gas exchange model, integrating the FvCB photosynthesis model and Ball-Berry stomatal conductance model, was combined with the node and leaf emergence model and leaf area expansion model to dynamically estimate rank-specific leaf area and canopy gas exchange.

The following sections detail the input variables for the simulation, the light extinction model used to calculate leaf rank-specific light intensity, the cropping type-specific and leaf-position-specific gas exchange modelling approach, and the calculation of photosynthate production and dry matter partitioning.

#### Input variables for simulation

The initial dry weight for the canopy photosynthesis and dry matter partitioning simulations were calculated by summing the leaf and stem dry weights measured prior to transplanting, along with the root dry weight, which was estimated by applying a root dry weight distribution factor of 11% [57], to the total dry weight of grafted cucumber seedlings reported in a previous study. Additionally, the initial node number (leaf number) was determined based on actual measurements from sampled plants, and the initial leaf area for each leaf was set by applying the estimated leaf area at thermal time zero.

The environmental variables used as input data for simulation model included light intensity at the top of the canopy (μmol m^−2^ s^−1^), relative humidity (%), and air temperature (°C). To minimize noise and irregular fluctuations, data collected at 1-minute intervals were aggregated into hourly averages for each hour of the day and used as input data for the simulation model.

To reflect the reduction in dry matter due to fruit harvest, the measured daily fruit harvest data (fresh weight) was first converted into dry weight using a dry weight conversion factor of 0.03 [58]. The resulting daily harvested fruit dry weight (g m^−2^) was incorporated into the model as an input variable. This preprocessed daily mean dry weight of harvested fruit was subsequently used to adjust the total plant dry matter in the simulation.

Details on the data collection methods for input variables provided in the Environmental, growth, and yield data section of data collection.

#### Light extinction model

The light extinction model, proposed by Monsi and Saeki [59], reflects the decrease in light intensity from the top to the bottom of the canopy due to shading by leaves, as shown in Equation (14).


(14) \begin{equation*} {I}_r={I}_0\times{e}^{-k\times{LAI}_{r-1}} \end{equation*}

where ${I}_r$ represents the light intensity (μmol m^−2^ s^−1^) at given leaf rank $r$, ${I}_0$ is the light intensity (μmol m^−2^ s^−1^) at the top of the canopy, $k$ is the extinction coefficient, which is 0.8 for cucumber [60, 61], and ${LAI}_{r-1}$ is the cumulative leaf area index up to the leaf rank $r-1$. Leaves ranks were assigned in ascending order from the top of the canopy downward, with the uppermost leaf designated as rank 1.

The cumulative leaf area index LAI was calculated as:


(15) \begin{equation*} { LA I}_r=\sum_{i=1}^r\frac{LA_i}{S} \end{equation*}

where $L{A}_i$ is the leaf area of *i*^th^ leaf, which was calculated using leaf expansion model, and $S$ is the ground surface area per plant (m^2^). Light absorption was calculated individually for each leaf rank, taking into account the shading effect from the leaves above.

The ${I}_r$ calculated for each leaf rank were used as input variables in the FvCB model, a submodel of coupled gas exchange model.

#### Cropping type-specific and leaf-position-specific coupled gas exchange model

Net and gross photosynthetic rates for each leaf were estimated using separately developed coupled gas exchange models for semi-forcing and forcing cropping types. Within each cropping type-specific model, leaves were classified into three positional classes (upper, middle, bottom) based on their rank, and the corresponding FvCB model specific to each class was applied to account for variations in photosynthetic capacity within the canopy.

Leaves ranked 1 to 5 were classified as the upper class and were modeled using ${\mathrm{FvCB}}_{\mathrm{upper}}$. Leaves ranked 6 to 10 were assigned to the middle class with ${\mathrm{FvCB}}_{\mathrm{middle}}$, while leaves ranked 11 or higher were categorized as the bottom class and modeled using ${\mathrm{FvCB}}_{\mathrm{bottom}}.$ This classification is expressed as follows:


(16) \begin{equation*} {\mathrm{FvCB}}_r=\left\{\begin{array}{c}{\mathrm{FvCB}}_{\mathrm{upper}},\kern0.75em 1\le \mathrm{r}\le 5\kern0.5em \\{}{\mathrm{FvCB}}_{\mathrm{middle}},\kern0.5em 6\le \mathrm{r}\le 10\\{}{\mathrm{FvCB}}_{\mathrm{bottom}},\kern2.25em \mathrm{r}\ge 11\end{array}\right. \end{equation*}

This approach ensured that each individual leaf absorbed light according to its rank-specific position, while its photosynthetic rate was determined based on the class-based gas exchange model.

#### Canopy photosynthesis, respiration and net assimilate production

To calculate daily canopy gross photosynthesis (*P*_gc_, g CO_2_ m^−2^ d^−1^), the gross photosynthetic rate for each rank, estimated using coupled gas exchange model, was multiplied by the corresponding leaf area per unit ground area specific to that rank. The resulting values, initially computed per second (μmol CO₂ m^−2^ s^−1^), were integrated over 24 hours to obtain the daily total (μmol CO₂ m^−2^ d^−1^). This was then converted to grams of CO_2_ per square meter per day using molar mass of CO_2_ (44.01 g mol^−1^).

Maintenance respiration (*R_m_*) was calculated by applying the required amount of CH_2_O for maintenance respiration, 0.015 per gram of dry matter (DM) at 20°C, using the temperature-dependent Q_10_ function [62], as described in Equation (17).


(17) \begin{equation*} {R}_m={Q_{10- value}}^{\left(\frac{T-20}{10}\right)}\times{R}_{m20} \end{equation*}

where *T* is air temperature in degrees Celsius, and the *Q*_10-value_ is 2 [62].

Daily net assimilate production from canopy photosynthesis and night respiration was calculated using Equation (18) [30].


(18) \begin{equation*} \Delta{W}_{\mathrm{cr}}=\frac{\frac{30}{44}\times{P}_{\mathrm{gc}}-{R}_m}{\mathrm{ASR}{\mathrm{Q}}_{\mathrm{cr}}} \end{equation*}

where Δ*W*_cr_ represents the rate or crop growth (g DM m^−2^ d^−1^), *P*_gc_ represents the crop’s gross photosynthesis (g CO_2_ m^−2^ d^−1^), and ASQR_cr_ denotes the assimilate requirement per gram of crop matter, with a value of 1.45 (g CH_2_O/g DW) [55].

On defoliation days, leaf dry weight was subtracted by multiplying the total leaf area of the defoliated leaves by the specific leaf area (SLA). The SLA values for the semi-forcing and forcing cropping types were 0.025 and 0.018 m^2^ g^−1^, respectively (our own data from this study). Similarly, the reduction in total plant dry matter due to fruit harvest was accounted for by deducting the daily harvested fruit dry weight provided as an input variable from the total plant dry matter in simulation.

#### Dry matter partitioning calculation

The daily partitioning ratio of dry matter to fruit was calibrated to determine the proportion of daily net assimilate production (*ΔW*_*cr*_) allocated to fruit. This calibration was performed using linear interpolation, based on the cultivation methods in these experiments, following the dry matter partitioning ratios reported by Marcelis, which account for the effects of temperature and the number of fruits per axil [63]. The specific equation used for calculating daily partitioning ratio of dry matter to fruit, as well as the derivation method based on linear interpolation, is detailed in Supplementary Information 5.

**Figure 6:**
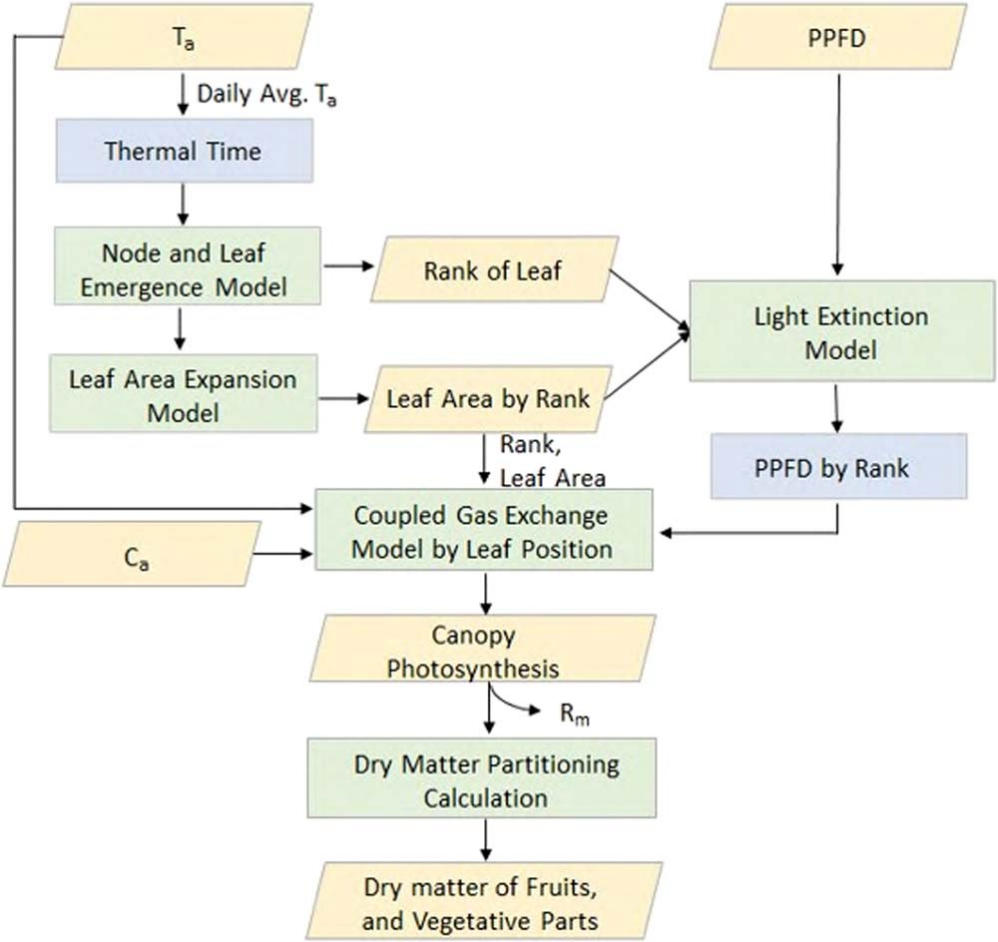
Schematic diagram of the canopy photosynthesis and dry matter partitioning simulation model.

A schematic diagram of the canopy photosynthesis and assimilate simulation model is provided in Fig. 6, and the code for the simulation model was written in Python language (see Supplementary Information 6 for the details).

#### Plant material and cultivation condition

From 2021 to 2024, one semi-forcing and three forcing cropping type experiments were conducted on cucumber (*Cucumis sativus*). In all experiments, ‘Baekdadagi’ cucumbers plants were grafted onto squash (*Cucurbita ficifolia* Bouche), transplanted into stonewool blocks (Saint-Gobain Cultilene BV, Netherlands), and subsequently planted into stonewool slabs (Saint-Gobain Cultilene BV, Netherlands) for the experiments. Detailed information regarding crop cultivars, experimental periods, plant density, and cropping type is provided in Table 5.

All experiments were conducted in a Venlo-type greenhouse at Kyungpook National University, Korea (35.5° N, 238.8° E; width, 8 m; length, 20 m; height, 7.4 m). The roof of the greenhouse was covered with anti-reflective glass, and the sidewalls were equipped with 16-mm polycarbonate panels, but lacked side ventilators. Plant densities were 1.72 plants m^−2^ in experiments 1 and 2, and 1.81 plants m^−2^ in experiments 3 and 4.

Roof vents, shading and thermal screens, and air source heat pumps integrated with thermal storage tanks were used for temperature control in both experiments. Irrigation was adjusted to 20%–30% drainage using drip systems based on accumulated radiation. The nutrient solution applied was the ‘Yamazaki’ cucumber standard nutrient solution [64]. In the initial phases, cucumber plants were supplied with a nutrient solution having an electrical conductivity (EC) of 1.0 dS·m^−1^. As plant growth progressed, the EC was gradually increased in increments of 0.2 dS·m^−1^. Upon reaching the generative growth phase, the EC of the nutrient solution was maintained at 2.4 dS·m^−1^ and the pH was regulated within the range of 5.8 to 6.0. Environmental conditions were managed autonomously using control software that operated according to predefined parameters (Synopta, Ridder, The Netherlands).

In all experiments, the number of leaves was maintained at approximately fifteen through regular defoliation. All lateral branches and shoots were pruned, leaving only the main stem intact. Tendrils were pruned. All flower below the 10^th^ node were removed, and from 10th node onwards, two flowers were retained per three nodes through flower thinning.

### Data collection

#### Environmental, growth and yield data

Air temperature (°C), relative humidity (RH, %), and humidity deficit (HD, g m^−3^) were measured using a dry/wet bulb sensor (MTV Active, Ridder, Netherlands). CO_2_ concentration was monitored using a CO_2_ sensor unit (MCO2, Ridder, Netherlands) and photosynthetically active radiation (PAR, W m^−2^) was recorded using a PAR sensor (Ridder Pyrgeometer, Ridder, Netherlands). Environmental data were collected at 1-minute intervals using a data logger (MultiMa, Ridder, Netherlands) positioned inside the greenhouse. This data was stored on a computer equipped with Synopta-HortiMax software.

Data on node numbers were collected on the day of transplanting by sampling 5 plants in experiments 1, 2, and 4 and 10 plants in experiment 3. Additionally, node number, leaf number, and yield data were collected after plant establishment by sampling 32, 23, 18, and 26 plants in experiments 1, 2, 3, and 4, respectively. Measurements of node and leaf numbers began 29, 30, 39, and 26 days after transplanting in experiments 1, 2, 3, and 4, respectively. The interval between measurements was one week for experiments 1 and 2 and two weeks for experiments 3 and 4. Yield data were collected daily in all experiments, with weight recorded in 5 g increments in experiments 1, 2, and 3 and 1 g units in experiment 4.

Leaf area was measured using destructive sampling methods at the end of each experiment. A total of 9, 15, 16, and 20 plants were sampled in experiments 1, 2, 3, and 4, respectively. The leaf area for each leaf, ranked from top to bottom, was measured using a leaf area meter (LI-3100C Area Meter, LI-COR Inc., USA) was used for the measurements.

#### Gas exchange measurement

To measure light and *A/C*_*i*_ responses under various environmental conditions, a photosynthesis system (LI-6400, LI-6400xt; LI-COR, USA) equipped with a red/blue LED light source attached to a 6 cm^2^ clamp-on leaf chamber was used. For each measurement, three cucumber plants were selected, and the 5^th^ (upper leaf), 10^th^ (middle leaf), and 15^th^ (bottom leaf) leaves, ranked from top to bottom, were selected for light and *A/C*_*i*_ response measurements. These measurements were conducted between 0800 and 1300 h to avoid the effects of afternoon photosynthesis depression.

*A/C*_*i*_ response curves were measured across eight levels of CO_2_ concentration. The incident photosynthetic photon flux density (PPFD) was maintained at 400 μmol m^−2^ s^−1^, the leaf chamber temperature at 25°C, the flow rate at 300 μmol s^−1^, and RH between 50% and 70%. The starting initial CO_2_ concentration was 400, followed by concentrations of 300, 200, 100, 50, 0, 400, 400, 600, 800, 1000, 1200, and 1500 μmol m^−2^ s^−1^. Data from the first and second measurements at 400 μmol m^−2^ s^−1^ were excluded from the analysis.

Light response curves were measured at seven levels of PPFD. The CO_2_ concentration in the leaf chamber was maintained at 400 μmol m^−2^ s^−1^, the leaf chamber temperature at 25°C, the flow rate at 300 μmol s^−1^, and RH was controlled between 50% and 70%. The initial PPFD was set at 1500 μmol m^−2^ s^−1^, followed by measurements at 1000, 800, 500, 250, 50, and 0 μmol m^−2^ s^−1^.

In the semi-forcing experiment (experiment 1), gas exchange measurements were conducted 19 times between 8 April 2021 and 2 September 2021, targeting the upper, middle, and bottom leaves. Similarly, in the forcing experiment (experiment 2), these measurements were conducted 22 times between 1 December 2021 and 26 April 2022, for leaves at all three positions (upper, middle, and bottom).

### Model calibration and validation

#### FvCB model calibration and validation

To calibrate the FvCB model for the upper, middle, and bottom in both the semi-forcing and forcing cropping types, 70% of the *A*/*C_i_* response was randomly samped wihin each cropping type and leaf position separately from gas exchange measurements in experiments 1 and 2, using the Scikit-learn library in Python.

The *J*_*max25*_*/V*_*cmax25*_ ratio was determined by analyzing *A*/*C_i_* data at a PPFD of 1500 μmol m^−2^ s^−1^, using the middle leaf from the forcing cropping type (experiment 3). The *θ* values were obtained using light response data for each cropping type and leaf position (see Supplementary Information 7 for the details) [65].

The SPSS statistical package (IBM, New York, NY, USA) was used to determine *V_c_* and *R*_d25_ values at a PPFD of 400 μmol m^−2^ s^−1^, utilizing data where *C_i_* was below 200 μmol m^−2^ s^−1^. The *V_c_* values at a PPFD of 400 μmol m^−2^ s^−1^, characterized by position and cropping type, were then fitted to the light intensity-dependent Rubisco activity function (Equation (S4–1) in Supplementary Information 4) to obtain *V*_*c*max25_ values. The calculated *V*_*c*max25_ values and the *J*_max25_/*V*_*c*max25_ ratio were used to determine *J*_max25_ values. The method used to the *J*_max25_/*V*_*c*max25_ ratio is described in Supplementary Information 8. The corresponding result is shown in Fig. S8 in Supplementary Information 8.

To validate the model, an independent dataset using the remaining 30% of the *A/Ci* response data that were not used for calibration, seperately for each cropping type and leaf position. Additionally, light reponse data measured in experiments 1 and 2 for each leaf position were included in validation dataset. This dataset was used only to evaluate the predictive performance of the model by comparing predicted and measured photosynthetic rates. Validation data were not used for parameter estimation, and data for calibration and validation were clearly separated to avoid circular validation.

#### Coupled gas exchange model validation

The validation dataset for coupled exchange model was identical to that used for the FvCB model validation in each cropping type and leaf position. Specifically, it consisted of *A/C*_*i*_ response data excluding the data used for FvCB model calibration, along with light response data for each cropping type and leaf position that were not used in calibration of FvCB model.

#### Node and leaf emergence model calibration and validation

The node and leaf emergence model was developed using data from experiment 1 and validated using data from experiments 2, 3, and 4.

#### Leaf expansion model calibration and validation

The leaf expansion model was constructed using leaf area data by rank, measured at the end of each experiment. The number of emergent leaves at the conclusion of the experiment calculated using the node and leaf emergence model, was used to determine the timing of emergence of each leaf rank. This allowed for the back-calculation of thermal time for each leaf rank. The model was calibrated with data from experiment 1 and validated using data from experiments 2, 3, and 4.

#### Photosynthetic and dry matter partitioning simulation model

To assess the performance of photosynthetic and dry matter partitioning simulations, we compared the leaf-position-specific model, which applies distinct FvCB parameters to upper, middle, and lower leaves, with the leaf-position-independent model, which applies the middle leaf FvCB parameters uniformly across all leaf positions.

### Statistical analysis

The models were evaluated using the coefficient of determination (*R*^2^, Equation (19)), and root mean square error (RMSE, Equation (20)), mean absolute error (MAE, Equation (21)), and bias (Equation (22)).


(19) \begin{equation*} {R}^2=1-\frac{\sum_{i=1}^n{\left({y}_i-{\hat{y}}_i\right)}^2}{\sum_{i=1}^n{\left({y}_i-{\overline{y}}_i\right)}^2} \end{equation*}

(20) \begin{equation*} \mathrm{RMSE}=\sqrt{\frac{1}{n}\sum_{i=1}^n{\left({y}_i-{\hat{y}}_i\right)}^2} \end{equation*}

(21) \begin{equation*} \mathrm{MAE}=\frac{1}{n}\sum_{i=1}^n\mid{y}_i-{\hat{y}}_i\mid \end{equation*}

(22) \begin{equation*} \mathrm{Bias}=\frac{1}{n}\sum_{i=1}^n\left({y}_i-{\hat{y}}_i\right) \end{equation*}

where *n* represents the number of observations, *y*_i_ refers to the actual observed values, ${\hat{\mathrm{y}}}_{\mathrm{i}}$represents the predicted values, and ${\overline{\mathrm{y}}}_{\mathrm{i}}$ is the mean of the actual observed values.

## Supplementary Material

Web_Material_uhaf124

## Data Availability

The data underlying this article are available in the article and in its online supplementary material.
