# Supplementary material for: Simulation of dry matter partitioning in cucumber fruits: reflecting gas exchange characteristics based on leaf position and cropping type
Source: Hortic Res. 2025 May 7;12(8):uhaf124. doi: 10.1093/hr/uhaf124 (PMC12261107; doi:10.1093/hr/uhaf124)
Supplement: Web_Material_uhaf124 [file web_material_uhaf124.zip › SUPPLE_1st revision_vf.pdf]

Supplementary information

Article title: Simulation of dry matter partitioning in cucumber fruits: reflecting gas exchange characteristics based on leaf position and cropping type

Authors: Ha Rang Shin[1†], Yu Hyun Moon[1†], Ha Seon Sim[1], Tae Yeon Lee[1], Soo Bin Jung[1], Yong Jun Kim[1], Na Kyoung Kim[1], Jin Woo Lee[1], Tae Hyun Kim[1], Seunghyun Ban[1*], Sung Kyeom Kim[1,2*]

The following Supporting Information is available for this article:

Supplementary information 1

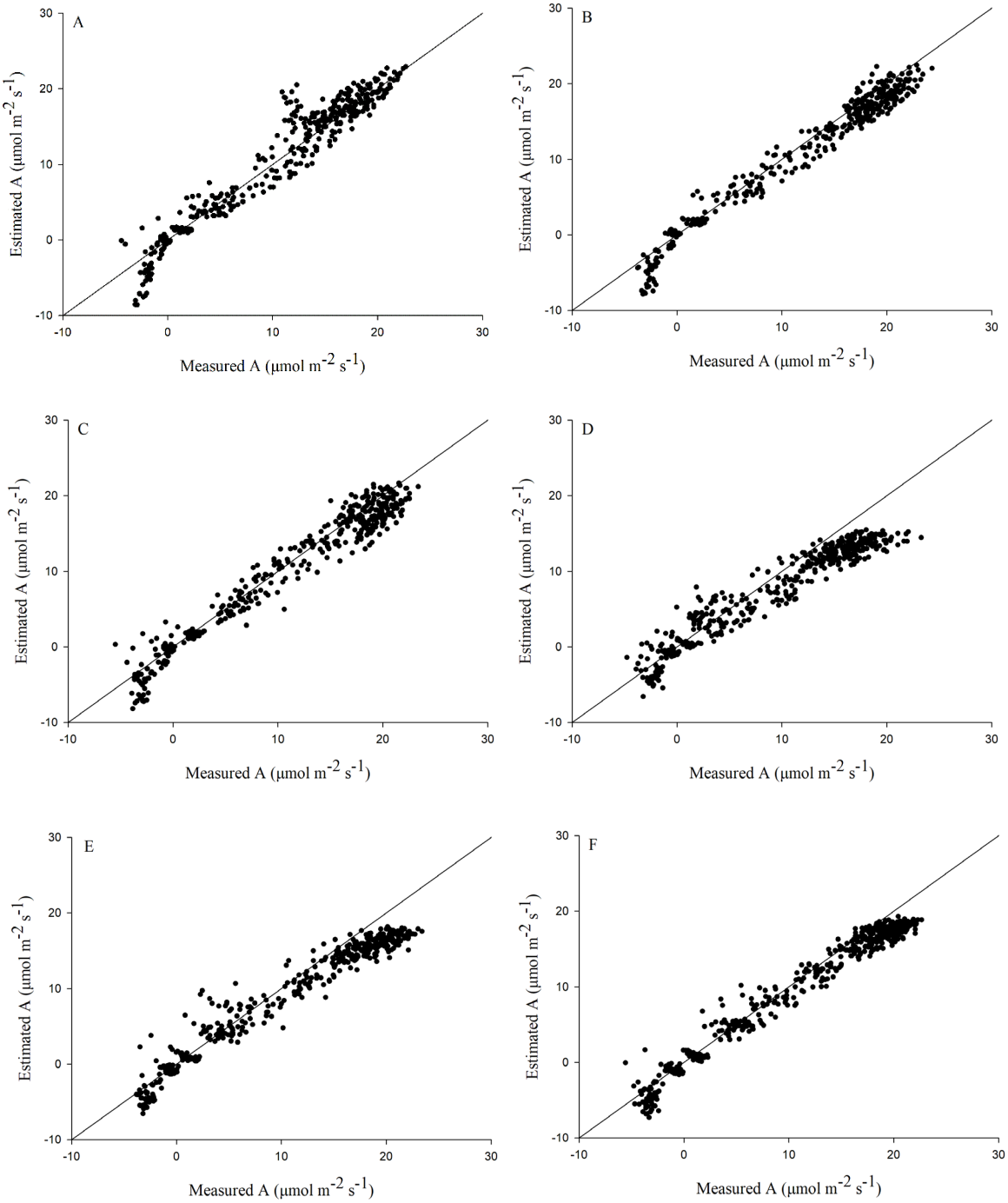

Figure S1A. Calibration results of the FvCB model for semi-forcing cropping type: A (upper leaves), B (middle leaves), C (bottom leaves). For forcing cropping type: D (upper leaves), E (middle leaves), F (bottom leaves).

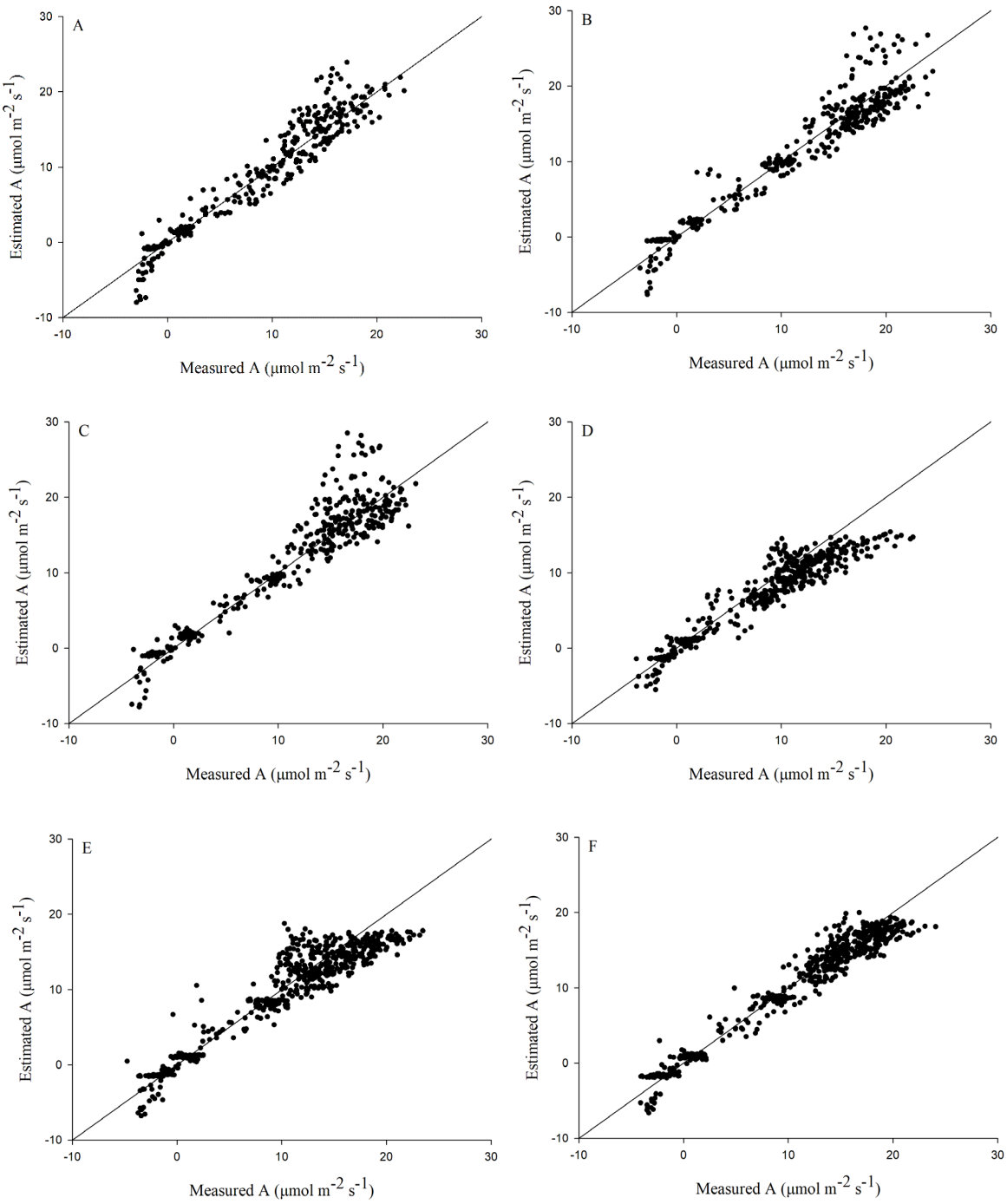

Figure S1B. Validation results of the FvCB model for semi-forcing cropping type: A (upper leaves), B (middle leaves), C (bottom leaves). For forcing cropping type: D (upper leaves), E (middle leaves), F (bottom leaves).

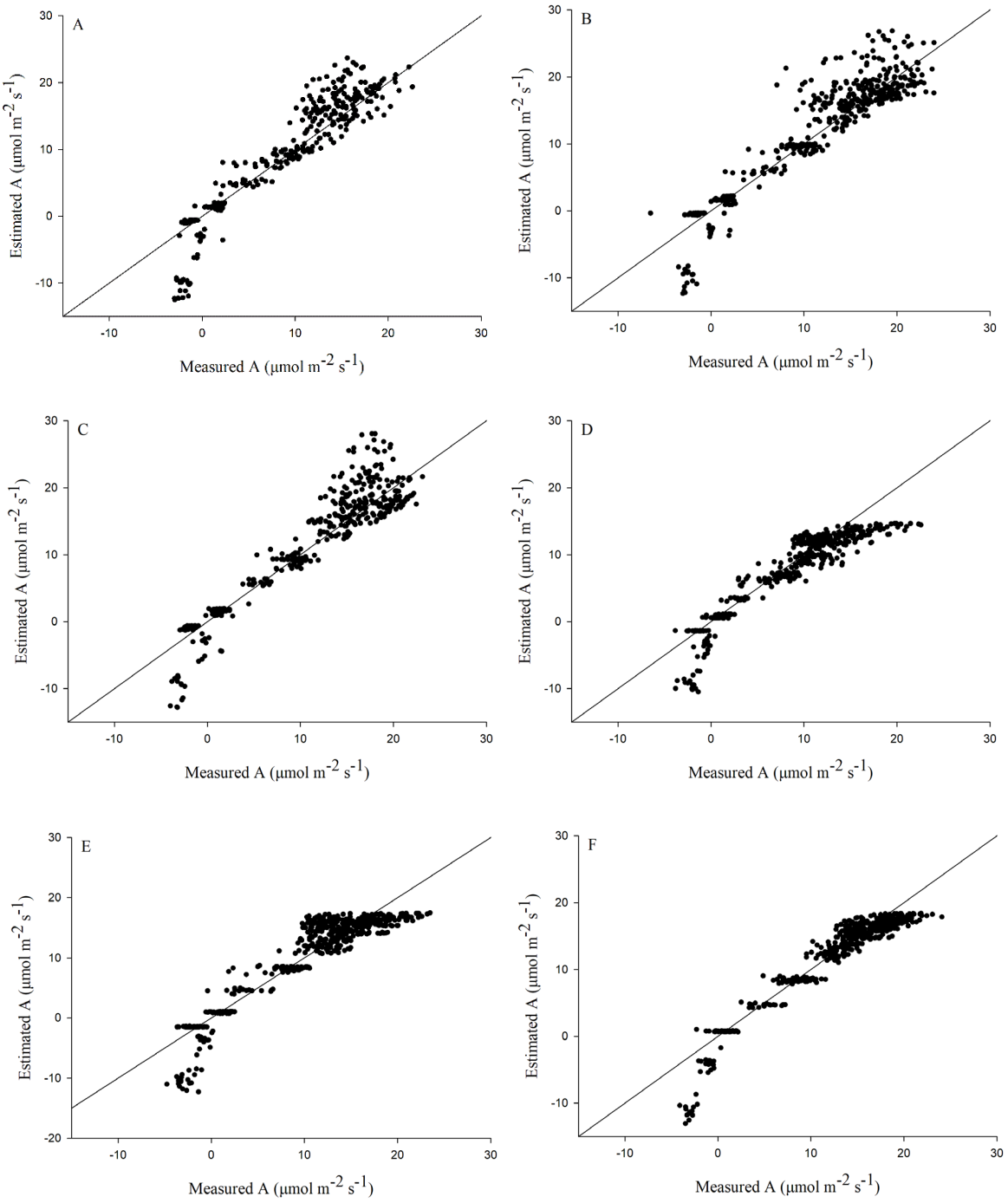

Figure S1C. Validation results of photosynthesis rate (A) using the coupled gas exchange model for semi-forcing cropping type: A (upper leaves), B (middle leaves), C (bottom leaves). For forcing cropping type: D (upper leaves), E (middle leaves), F (bottom leaves).

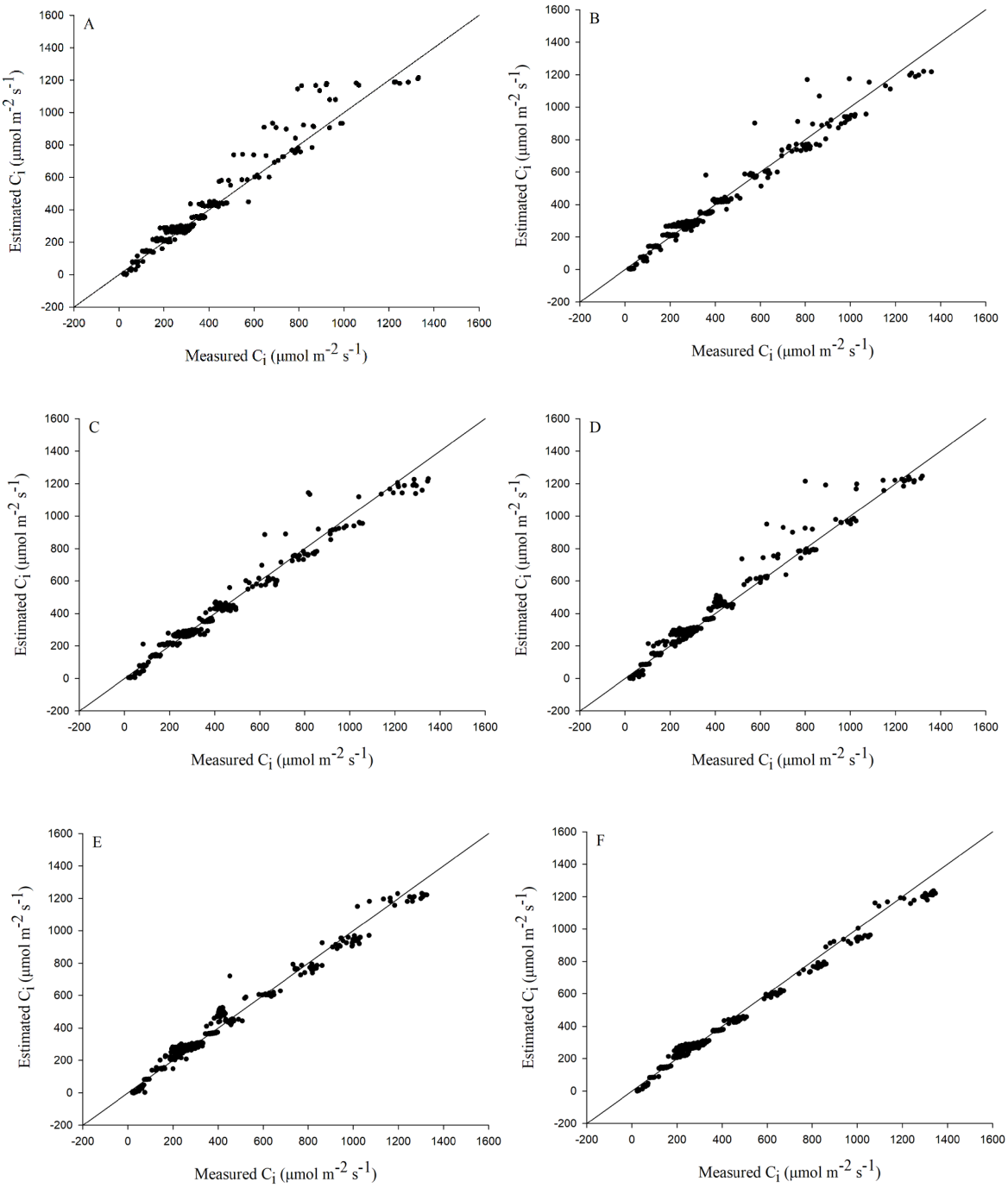

Figure S1D. Validation results of intercellular CO_2 concentration (C_i) using the coupled gas exchange model for semi-forcing cropping type: A (upper leaves), B (middle leaves), C (bottom leaves). For forcing cropping type: D (upper leaves), E (middle leaves), F (bottom leaves).

Supplementary information 2

Marcelis (1994) reported the ratio of root dry weight to total vegetative dry weight (including leaves, stem, petioles, and root) under different temperature conditions and fruit-to-lear ratios (x/y). According to this study, when x/y represents the number of fruit per leaf, the root dry weight to total vegetative dry weight ratios at 18°C were 0.18, 0.16, 0.16, and 0.14 for x/y values of 0, 1/6, 1/3, and 1/1, respectively. At 25°C, the corresponding ratios were 0.062, 0.060, 0.063, and 0.065 for the same x/y values (Marcelis 1994).

Using linear interpolation, the root dry weight to total vegetative dry weight ratio at x/y = 2/3 was estimated to be 0.15 at 18°C and 0.064 at 25°C. Based on this, we derived a linear equation (Eqn. S2-1) that describes the root dry weight to total vegetative dry weight ratio as a function of average air temperature when x/y = 2/3. Note that in Eqn. S2-1, the symbol "×" represents multiplication, not the x variable from the fruit-to-leaf ratio (x/y).

$$Root\ dry\ weight\ to\ total\ vegetative\ dry\ weigth\ ratio = -0.01229 \times T_{avg} + 0.3722 \quad \text{(Eqn. S2-1)}$$

Supplementary information 3

Table S1. Key modifications to adapt the simulation code from cucumber to other crops organized by relevant code sections and parameters

Category	Key Cahnges	Where in code
1. Data Input & Initialization	- Excel file path & sheet name updates<br>- Column renaming (temperature, PARi, DateTime)<br>- Adjustment of start_date	SimulationInitializer
2. Leaf Growth Logic	- Thermal time thresholds<br>- Base temperature/heat-unit parameters<br>- Gompertz parameters (a, b, c)<br>- Leaf-removal logic	LeafCalculator
3. Photosynthesis Model	- New Vcmax_25, Jmax_25, Rd_25 defaults<br>- Temperature response parameters	FvCB_Calculator
4. Stomatal Conductance	- CO_2, humidity, VPD references<br>- update_Ci_and_recalculate modifications	FvCB_Calculator
5. Partitioning & Respiration	- New partition ratios (e.g., vegetative vs. reproductive)<br>- Respiratory coefficients<br>- Adjustments to daily partition logic	simulate_photosynthesis
6. Fruit or Organ Growth	- fruit_dw_file_path replacement<br>- Hourly/daily assimilation to fruit vs. vegetative	simulate_photosynthesis (final)
7. Species-Specific Configuration	- Central config file or dictionary for crop parameters<br>- Dynamic referencing in main classes	Separate config or constructor
8. Calibration & Validation	- Experimental data gathering or re-analysis<br>- Comparison with field measurements<br>- Iterative parameter tuning	Outside main code
9. Additional Environment or Management	- Adaptation to drastically different conditions (paddy, orchard, greenhouse)<br>- Additional stress functions (drought, salinity)	Potentially all classes

Supplementary information 4

According to the light intensity-dependent model of Rubisco activation in previous studies, it increases in a logarithmic function starting at 31% at light intensity 0 (μmol m^{-2} s^{-1}) (Qian *et al.* 2012). The data used to calibrate the light intensity-dependent Rubisco activity model are A/C_i data measured 17 times each at PPFD 400, 800, and 1500 (μmol m^{-2} s^{-1}) from December 3, 2021 to April 21, 2022, using mid-position leaves of Experiment 2, *'Hangangmatbaekdadagi'* as material. The method of measuring the A/C_i curve is the same except for the light intensity set in the Materials and Methods of this paper. The V_c values at each light intensity were calculated using SPSS (IBM) and found to be 49.37, 66.71, and 70.85 for PPFD 400, 800, and 1500 (μmol m^{-2} s^{-1}) respectively. The methodology for estimating V_c values is the same as in the main text. Assuming that the V_c value for PPFD 1500 (μmol m^{-2} s^{-1}) is V_{cmax25}, i.e. 100% Rubisco activation (Qian et al. 2012), the Rubisco activation for PPFD 400 and 800 (μmol m^{-2} s^{-1}) is 69.68% and 94.15%, respectively. The results of calibrating a light intensity-dependent model of Rubisco activation based on activity at zero light intensity from the original model are shown in Eqn. S4-1 and Fig. S4.

$$V_c = V_{cmax}\left(\frac{31+\frac{69}{\exp\left(-0.005\ (PPFD-350)\right)}}{100}\right) \quad \text{(S4-1)}$$

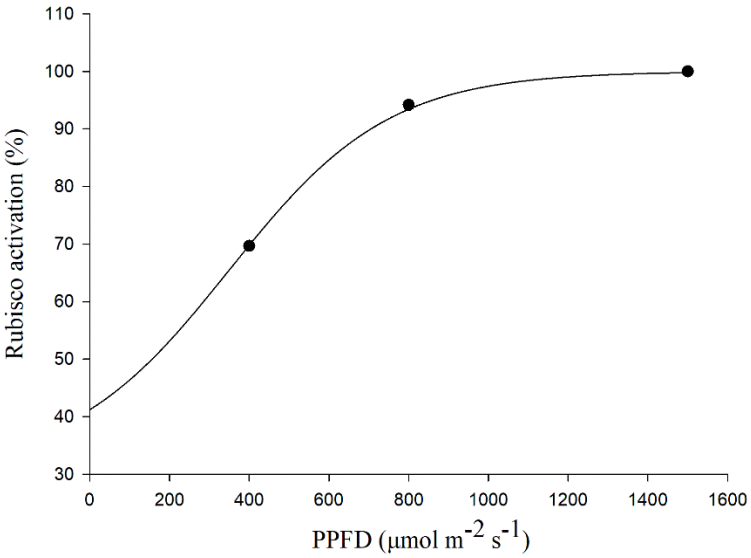

Figure S4. Rubisco activation dependence on light intensity.

Supplementary information 5

Marcelis (1993) reported the dry matter partitioning ratio to fruit, defined as the ratio of cumulative fruit dry weight to total plant dry weight under different temperature conditions and fruit-to-leaf ratios (x/y). According to this study, when x/y represents the number of fruits per leaf, the partitioning ratios at 18°C were 0, 0.25, 0.38, and 0.48 for x/y values of 0, 1/6, 1/3, and 1/1, respectively, and at 25°C, the partitioning ratios were 0, 0.35, 0.45, and 0.52 for the same x/y values (Marcelis, 1993).

Using linear interpolation, the partitioning ratio at x/y = 2/3 was estimated to be 0.43 at 18°C and 0.485 at 25°C. Based on this, we derived a linear equation (Eqn. S5-1) that describes the fraction of total plant dry matter allocated to fruits as a function of average air temperature when x/y = 2/3. Note that in Eqn. S2-1, the symbol "×" represents multiplication, not the x variable from the fruit-to-leaf ratio (x/y).

This equation was then used to calculate the daily partitioning ratio of dry matter allocated to fruits, which represents the proportion of the total dry matter produced each day that is allocated to fruits.

Daily partitioning ratio of dry matter to fruits (PF(T)) = 0.0786 × T + 0.2886 (Eqn. S5-1)

where T is the daily average air temperature (°C), and PF(T) represents the fraction of daily dry matter partitioned to fruits.

Supplementary information 6

[Python code of simulation model]

Variables set at startup

Both cumulative_sum and cumulative_thermal_time are initialized to zero. Variables such as nodes (number of nodes), retaining_leaves (number of leaves remaining), and leaf_number (number of leaves) are set the same as initial_nodes. The resulting DataFrame with an index over time during the simulation period is created, which stores, among other things, the accumulated column time up to each time and including that time.

Initialize leaf information

For each initial node (from 1 to initial_nodes), an entry containing the leaf number, simulation start date, and initial column time 0 is added to the leaves_info list.

Daily loop for ten hours accumulation

The method repeats the following steps for each date (indexed to daily_avg_temp).

Extract daily parameters:
Current Date is the current iteration date. Daily Growth Temperatures is the value read from daily_avg_temp ['daily_growing_temp'] for the current date. This is the result of subtracting 10 from the average daily temperature.

Determining column thresholds

Thresholds for leaf appearance are selected based on the current date. If the current date is earlier than split_date, threshold_before is used. Otherwise, threshold_after is used.

Accumulating thermal time

Daily_growing_temp (daily growing temperature) is added to the cumulative_sum. When cumulative_sum exceeds the threshold, it indicates that the cucumber has accumulated enough thermal time to generate a new node. Then it increases nodes, retaining_leaves, and leaf_number. Subtract the threshold value from the cumulative_sum (as a result, the accumulation towards the next node is "reset"). Add a new entry to leaves_info that includes the number of the new leaf, the current date, and the current cumulative thermal time (before the temperature of that date is added).

Hourly Interpolation of Thermal Time

Time-specific time ranges are configured for the current date. For each time in this range: cumulative_thermal_time_until_yesterday is set to the cumulative_thermal_time value. The first time of the simulation is explicitly set to zero. The cumulative_thermal_time for that time is calculated as the sum of the cumulative_thermal_time and the daily_growing_temp for that date. After processing all the time of the day, update the entire cumulative_thermal_time by adding the daily_growing_temp of the current date.

Leaf removal process

Separately, if the current date matches one of the leaf removal schedules, the process of pruning the leaves begins (if the number of leaves exceeds 15). This step is integrated within the daily

loop and uses hourly time series to determine the exact moment of leaf removal.

Repeated C_i updates using Newton-Raphson approach

The initial C_i is set based on the external CO_2 concentration. The FvCB model calculates the A, which is used in Ball-Berry models to update the g_s and to repeatedly fine-tune the C_i. This process is repeated until C_i is stabilized.

Convergence criteria

Repeated updates are interrupted when $|\text{new } C_i - \text{initial } C_i| \leq 0.001$. Limit the maximum number of iterations to 100 to end even if they do not converge.

Damped Iterative Updates

Instead of drastic changes, C_i is gradually updated to prevent vibration.

Fallback Handling

If the number of iterations exceeds 100, the process stops and a warning message is output. These techniques, implemented in the update_Ci_and_recalculate() function, ensure numerical stability while calculating C_i, A, and g_s and prevent infinite loops (a phenomenon in which the program continues to repeat without ending).

Simulation model code

```
import pandas as pd
import numpy as np
import matplotlib.pyplot as plt


# Define the Gompertz growth function
def gompertz_growth(t, a, b, c):
    return a * np.exp(-np.exp(-(t - b) / c))


class SimulationInitializer:
    def __init__(self, file_path, sheet_name, start_date, initial_nodes, threshold_before, threshold_after, split_date,
leaf_removal_dates_file, leaf_removal_sheet_name):
        self.file_path = file_path
        self.sheet_name = sheet_name
        self.start_date = start_date
        self.initial_nodes = initial_nodes
        self.threshold_before = threshold_before
        self.threshold_after = threshold_after
        self.split_date = split_date
```

```
167             self.leaf_removal_dates_file = leaf_removal_dates_file
168             self.leaf_removal_sheet_name = leaf_removal_sheet_name
169
170         def initialize(self):
171             df = pd.read_excel(self.file_path, sheet_name=self.sheet_name)
172             df['DateTime'] = pd.to_datetime(df['DateTime'])
173             df.set_index('DateTime', inplace=True)
174             df.rename(columns={'Air temperature': 'temperature', 'PPF': 'PARi'}, inplace=True)
175             df = df[df.index >= self.start_date]
176             df_hourly = df.resample('H').mean()
177             daily_avg_temp = df_hourly.resample('D').mean()
178             daily_avg_temp['daily_growing_temp'] = daily_avg_temp['temperature'] - 10
179             daily_avg_temp.index = daily_avg_temp.index.date    # Convert index to datetime.date format
180
181             # Load leaf removal dates with sheet name
182             leaf_removal_dates = pd.read_excel(self.leaf_removal_dates_file, sheet_name=self.leaf_removal_sheet_name)
183             leaf_removal_dates['Date'] = pd.to_datetime(leaf_removal_dates['Date']).dt.date
184
185             return df, df_hourly, daily_avg_temp, leaf_removal_dates
186
187 class LeafCalculator:
188         def __init__(self, df_hourly, daily_avg_temp, initial_nodes, threshold_before, threshold_after, split_date,
189 leaf_removal_dates, a, b, c, conversion_factor):
190             self.df_hourly = df_hourly
191             self.daily_avg_temp = daily_avg_temp
192             self.initial_nodes = initial_nodes
193             self.threshold_before = threshold_before
194             self.threshold_after = threshold_after
195             self.split_date = split_date
196             self.leaf_removal_dates = leaf_removal_dates
197             self.a = a
198             self.b = b
199             self.c = c
200             self.conversion_factor = conversion_factor
201
202         def calculate_leaves_and_fill_hourly(self):
```

```
            cumulative_sum = 0
            nodes = self.initial_nodes
            remaining_leaves = self.initial_nodes
            leaf_number = self.initial_nodes
            leaves_info = []
            removed_leaves_info = []
            cumulative_thermal_time = 0
            global result
            result = pd.DataFrame(index=pd.date_range(start=self.df_hourly.index.min(), end=self.df_hourly.index.max(),
freq='H'))
            result['remaining_leaves'] = np.nan
            result['total_nodes'] = np.nan
            result['cumulative_thermal_time_until_yesterday'] = np.nan
            result['cumulative_thermal_time'] = np.nan
            simulation_start_date = self.df_hourly.index[0]
            for leaf_number in range(1, self.initial_nodes + 1):
                leaves_info.append({'Leaf Number': leaf_number, 'Date': simulation_start_date, 'Thermal Time': 0})

            for i in range(len(self.daily_avg_temp)):
                current_date = self.daily_avg_temp.index[i]
                daily_growing_temp = self.daily_avg_temp['daily_growing_temp'].iloc[i]
                threshold = self.threshold_before if current_date < pd.to_datetime(self.split_date).date() else
self.threshold_after
                cumulative_sum += daily_growing_temp
                if cumulative_sum > threshold:
                    nodes += 1
                    remaining_leaves += 1
                    cumulative_sum -= threshold
                    leaf_number += 1
                    leaves_info.append({'Leaf Number': leaf_number, 'Date': current_date, 'Thermal Time':
cumulative_thermal_time})

                start_time = current_date
                end_time = start_time + pd.Timedelta(days=1)
                times = pd.date_range(start_time, end_time, freq='H')[:-1]
                for current_time in times:
                    if i == 0 and current_time == times[0]:
```

```
                    result.loc[current_time, 'cumulative_thermal_time_until_yesterday'] = 0
                else:
                    result.loc[current_time, 'cumulative_thermal_time_until_yesterday'] = cumulative_thermal_time
                result.loc[current_time, 'cumulative_thermal_time'] = cumulative_thermal_time + daily_growing_temp
            cumulative_thermal_time += daily_growing_temp

            # Leaf removal process based on provided dates
            if current_date in self.leaf_removal_dates['Date'].values:
                # Ensure that the 'Time' column is of datetime type and then convert it to string
                self.leaf_removal_dates['Time']=pd.to_datetime(self.leaf_removal_dates['Time'],
format='%H:%M:%S').dt.time
                removal_time_str = self.leaf_removal_dates.loc[self.leaf_removal_dates['Date']==current_date,
'Time'].astype(str).values[0]
                removal_time = pd.Timestamp(f"{current_date} {removal_time_str}").time()
                for current_time in times:
                    if current_time.time() < removal_time:
                        result.loc[current_time, 'remaining_leaves'] = remaining_leaves
                        result.loc[current_time, 'total_nodes'] = nodes
                    if current_time.time() == removal_time:
                        if remaining_leaves > 15:
                            leaves_to_remove = remaining_leaves - 15
                            remaining_leaf_indices = list(range(len(leaves_info) - remaining_leaves,
len(leaves_info)))
                            leaves_to_prune_indices = remaining_leaf_indices[:leaves_to_remove]
                            removed_leaves = [leaves_info[i] for i in leaves_to_prune_indices]
                            removed_leaf_area = sum(gompertz_growth(result.loc[current_time,
'cumulative_thermal_time'] - leaf['Thermal Time'], self.a, self.b, self.c) for leaf in removed_leaves)
                            removed_leaf_area_m2 = removed_leaf_area * self.conversion_factor
                            removed_leaves_info.append({
                                'Date': current_date,
                                'Removed Leaves': [leaf['Leaf Number'] for leaf in removed_leaves],
                                'Removed Leaf Area': removed_leaf_area,
                                'Removed Leaf Area_m2': removed_leaf_area_m2
                            })
                            remaining_leaves = 15
                    if current_time.time() >= removal_time:
                        result.loc[current_time, 'remaining_leaves'] = remaining_leaves
```

```
                                result.loc[current_time, 'total_nodes'] = nodes
                    else:
                        for current_time in times:
                            result.loc[current_time, 'remaining_leaves'] = remaining_leaves
                            result.loc[current_time, 'total_nodes'] = nodes

            return result, leaves_info, removed_leaves_info

        def create_remaining_leaves_info(self, result_combined, leaves_info_df):
            remaining_leaves_info = []
            for i in range(len(result_combined)):
                row = result_combined.iloc[i]
                remaining_leaves_count = row['remaining_leaves']
                cumulative_thermal_time_until_yesterday = row['cumulative_thermal_time_until_yesterday']
                if not np.isnan(remaining_leaves_count):
                    timestamp = row.name
                    leaves_info_df['Date'] = pd.to_datetime(leaves_info_df['Date'])    # Ensure that 'Date' is datetime
                    current_leaves_info = leaves_info_df[leaves_info_df['Date'] <=
timestamp].tail(int(remaining_leaves_count))[['Leaf Number', 'Thermal Time']].copy()
                    current_leaves_info = current_leaves_info.iloc[::-1]
                    current_leaves_info['Rank'] = range(1, len(current_leaves_info) + 1)
                    current_leaves_info['Timestamp'] = timestamp
                    current_leaves_info['Leaf Area'] = current_leaves_info.apply(lambda x:
gompertz_growth(cumulative_thermal_time_until_yesterday - x['Thermal Time'], self.a, self.b, self.c), axis=1)
                    current_leaves_info['Leaf_Area_per_m2'] = current_leaves_info['Leaf Area'] * self.conversion_factor
                    remaining_leaves_info.append(current_leaves_info)
            return remaining_leaves_info

        def transform_remaining_leaves_info(self, remaining_leaves_info):
            transformed_data = []
            for timestamp, group in pd.concat(remaining_leaves_info).groupby('Timestamp'):
                row = [timestamp]
                for _, leaf_info in group.iterrows():
                    row.extend([leaf_info['Leaf Number'], leaf_info['Thermal Time'], leaf_info['Leaf Area'],
leaf_info['Leaf_Area_per_m2']])
                transformed_data.append(row)
            return transformed_data
```

```
314
315         def pad_transformed_data(self, transformed_data):
316             max_len = max(len(row) for row in transformed_data)
317             for row in transformed_data:
318                 row.extend([None] * (max_len - len(row)))
319             return transformed_data, max_len
320
321         def create_columns(self, max_len):
322             columns = ['Timestamp']
323             for i in range(1, (max_len - 1) // 4 + 1):
324                 columns.extend([f'Leaf_Rank_{i}', f'Thermal_Time_{i}', f'Leaf_Area_{i}', f'Leaf_Area_per_m2_{i}'])
325             return columns
326     # You need to provide the values for FvCB model parameters
327     class FvCB_Calculator:
328         def __init__(self, df, final_leaves_info_df):
329             self.df1 = df
330             self.final_leaves_info_df = final_leaves_info_df
331             self.default_Vcmax_25 = 77.91 ##3:71.98    2: 77.91
332             self.default_Jmax_25 = 132.45    ##3:122.37 2:132.45
333             self.default_Rd_25 = 0.63          ##3: 1.61    2:0.63
334             self.T = self.df1['temperature']
335             self.Ci = self.df1['Ci']
336             self.PARi = self.df1['PARi']
337             self.Vcmax_25 = self.default_Vcmax_25
338             self.Jmax_25 = self.default_Jmax_25
339             self.Rd_25 = self.default_Rd_25
340
341         def set_params_by_rank(self, rank):
342             if rank <= 5:
343                 self.Vcmax_25 = self.default_Vcmax_25
344                 self.Jmax_25 = self.default_Jmax_25
345                 self.Rd_25 = self.default_Rd_25
346                 self.theta =    0.71
347             elif rank <= 10:
348                 self.Vcmax_25 = 74.35
349                 self.Jmax_25 = 126.40
```

```
                self.Rd_25 = 0.35
                self.theta = 0.76
            else:
                self.Vcmax_25 = 63.82
                self.Jmax_25 = 108.50
                self.Rd__25 = 0.56
                self.theta = 0.88

    def calculate_PPF_REAL(self, i, rank):
        PARi = self.df1.iloc[i]['PARi']
        SUM_RANK_LAI = sum(
            self.final_leaves_info_df.loc[self.final_leaves_info_df['Timestamp'] == self.df1.index[i],
f'Leaf_Area_per_m2_(Kalyanaraman et al.)'].values[0]
            for j in range(1, rank + 1)
            if not np.isnan(self.final_leaves_info_df.loc[self.final_leaves_info_df['Timestamp'] == self.df1.index[i],
f'Leaf_Area_per_m2_(Kalyanaraman et al.)'].values[0])
        )
        PPF_REAL = PARi * np.exp(-0.8 * SUM_RANK_LAI)
        return PPF_REAL

    def calculate_A(self, i, rank):
        T = self.T.iloc[i]
        Ci = self.df1.iloc[i]['Ci']
        PARi = self.calculate_PPF_REAL(i, rank)
        V_Ha = 91185
        R = 8.314
        V_S = 650
        V_Hd = 202900
        J_Ha = 79500
        J_S = 650
        J_Hd = 201000
        O = 210
        a = 0.3
        theta = self.theta

        Vcmax_25 = self.Vcmax_25
        Jmax_25 = self.Jmax_25
```

```
            Rd_25 = self.Rd_25

            T_K = T + 273.15    # Convert Celsius to Kelvin
            gammastar = 42.75 * np.exp(37830 * (T_K - 298) / (298 * R * T_K))
            Kc = 404.9 * np.exp(79430 * (T_K - 298) / (298 * R * T_K))
            Ko = 278.4 * np.exp(36380 * (T_K - 298) / (298 * R * T_K))
            Rd = Rd_25 * 2 ** ((T - 25) / 10)

            Vc = (Vcmax_25 * ((31 + (69 / (1 + np.exp(-0.005 * (PARi - 350))))) / 100) *
                  np.exp(V_Ha * (T - 25) / ((25 + 237.15) * R * T_K)) *
                  ((1 + np.exp((V_S - V_Hd) / ((25 + 273.15) * R))) /
                   (1 + np.exp((V_S - V_Hd) / (T_K * R)))))
            A1 = (Vc * (Ci - gammastar) / (Ci + Kc * (1 + O / Ko))) - Rd

            Jmax = (Jmax_25 * np.exp(J_Ha * (T - 25) / ((25 + 237.15) * R * T_K)) *
                    ((1 + np.exp((J_S - J_Hd) / ((25 + 273.15) * R))) /
                     (1 + np.exp((J_S - J_Hd) / (T_K * R)))))
            J = (a * PARi + Jmax - np.sqrt((a * PARi + Jmax) ** 2 - 4 * theta * a * PARi * Jmax)) / (2 * theta)
            A2 = (J * (Ci - gammastar) / (4 * Ci + 8 * gammastar)) - Rd

            if np.isnan(A1) and np.isnan(A2):
                print(f"NaN detected in both A1 and A2 at Row: {i}, Rank: {rank}")
                return None, None

            if A1 < A2:
                A = A1
            else:
                A = A2

            return A, Rd

        def calculate_gsc_gsw(self, i, A):
            h = self.df1.iloc[i]['hs']
            CO2 = self.df1.iloc[i]['CO2']
            gsw = ((A * 8.376 * h) / CO2) + 0.045
            gsc = gsw / 1.6
```

```
423
424                 return gsc, gsw
425
426         def update_Ci_and_recalculate(self, i, rank):
427                 self.set_params_by_rank(rank)
428                 Ci_initial = self.df1.iloc[i]['Ci']
429                 iteration = 0 # Iteration count variable for debugging
430                 while True:
431                         A, Rd = self.calculate_A(i, rank)
432                         if A is None:
433                                 return None    # return None if NaN is encountered
434
435                         gsc, gsw = self.calculate_gsc_gsw(i, A)
436                         Ci_new = self.df1.iloc[i]['CO2'] - A / gsc
437
438                         if abs(Ci_new - Ci_initial) <= 0.001 or iteration > 100:
439                                 if iteration > 100:
440                                         print(f"Max iterations reached for row {i}, Rank: {rank}")
441                                 return A, gsc, gsw, Ci_new, Rd
442
443                         self.df1.at[self.df1.index[i], 'Ci'] = Ci_new
444                         Ci_initial = Ci_new
445                         iteration += 1
446
447 def calculate_photosynthesis_rates(df_combined, final_leaves_info_df):
448         calculator = FvCB_Calculator(df_combined, final_leaves_info_df)
449         results = []
450         total_photosynthesis_by_time = []
451
452         for i in range(len(df_combined)):
453                 timestamp = df_combined.index[i]
454                 total_gross_photosynthesis_amount = 0
455
456                 remaining_leaves_count = df_combined.iloc[i]['remaining_leaves']
457                 if np.isnan(remaining_leaves_count):
458                         continue
```

```
459
460             for rank in range(1, int(remaining_leaves_count) + 1):
461                 leaf_area_per_m2 = final_leaves_info_df.loc[final_leaves_info_df['Timestamp'] == timestamp,
462 f'Leaf_Area_per_m2_{rank}'].values
463                 if len(leaf_area_per_m2) == 0:
464                     continue
465                 leaf_area_per_m2 = leaf_area_per_m2[0]
466
467                 calculation_result = calculator.update_Ci_and_recalculate(i, rank)
468                 if calculation_result is None:
469                     continue
470
471                 A, gsc, gsw, Ci_new, Rd = calculation_result
472                 PPF_REAL = calculator.calculate_PPF_REAL(i, rank)
473                 gross_A = A + Rd
474                 rank_gross_photosynthesis_rate = gross_A * leaf_area_per_m2
475                 rank_gross_photosynthesis_amount = rank_gross_photosynthesis_rate * 3600    # photosynthesis for 1 hour
476                 total_gross_photosynthesis_amount += rank_gross_photosynthesis_amount
477
478                 results.append({
479                     'Timestamp': timestamp,
480                     'Rank': rank,
481                     'gross_A': gross_A,
482                     'rank_gross_photosynthesis_rate/hr': rank_gross_photosynthesis_rate,
483                     'rank_gross_photosynthesis_amount/hr': rank_gross_photosynthesis_amount,
484                     'PPF_REAL': PPF_REAL
485                 })
486
487             total_photosynthesis_by_time.append({
488                 'Timestamp': timestamp,
489                 'total_gross_photosynthesis_amount/hr': total_gross_photosynthesis_amount
490                 })
491
492         return pd.DataFrame(results), pd.DataFrame(total_photosynthesis_by_time)
493
494 def calculate_rm(result_combined):
```

```
        daily_avg_temp = result_combined.resample('D').mean()['temperature']
        daily_avg_temp.index = daily_avg_temp.index.date    # Convert index to datetime.date format

        # Calculate Rm_Vegetative and Rm_Fruit
        rm_vegetative = 0.033 * (2 ** ((daily_avg_temp - 25) / 10))
        rm_fruit = 0.015 * (2 ** ((daily_avg_temp - 25) / 10))

        rm_df = pd.DataFrame({
            'Date': daily_avg_temp.index,
            'Rm_Vegetative (CH2Og/g DM)': rm_vegetative.values,
            'Rm_Fruit (CH2Og/g DM)': rm_fruit.values
        })

        return rm_df

def add_fruit_dw_column(daily_photosynthesis_df, fruit_dw_file_path, fruit_dw_sheet_name):
        fruit_df = pd.read_excel(fruit_dw_file_path, sheet_name=fruit_dw_sheet_name)
        fruit_df = fruit_df[['Date', 'Fruit DW/m^2']]
        fruit_df['Date'] = pd.to_datetime(fruit_df['Date']).dt.date

        daily_photosynthesis_df['Date'] = pd.to_datetime(daily_photosynthesis_df['Date']).dt.date

        daily_photosynthesis_df = daily_photosynthesis_df.merge(fruit_df, on='Date', how='left')
        daily_photosynthesis_df['Fruit DW/m^2'].fillna(0, inplace=True)
        daily_photosynthesis_df.rename(columns={'Fruit DW/m^2': 'Harvested Fruit DW (g/m^2)'}, inplace=True)

        return daily_photosynthesis_df

def simulate_photosynthesis(file_path, sheet_name, start_date, initial_nodes, threshold_before, threshold_after, split_date,
                            leaf_removal_dates_file, leaf_removal_sheet_name, output_file_path, SLA,
fruit_dw_file_path,
                            fruit_dw_sheet_name, partitioning_vegetative_before, partitioning_fruit_before,
                            initial_remaining_vegetative_dw, plant_density_per_m2):
        initializer = SimulationInitializer(file_path, sheet_name, start_date, initial_nodes, threshold_before, threshold_after,
split_date, leaf_removal_dates_file, leaf_removal_sheet_name)
        df, df_hourly, daily_avg_temp, leaf_removal_dates = initializer.initialize()

```

```
        # You need to provide the values for a, b, and c
        a, b, c = 582.06, 45.33, 45.31
        conversion_factor = plant_density_per_m2 / 10000

        leaf_calculator = LeafCalculator(df_hourly, daily_avg_temp, initial_nodes, threshold_before, threshold_after,
split_date, leaf_removal_dates, a, b, c, conversion_factor)
        result, leaves_info, removed_leaves_info = leaf_calculator.calculate_leaves_and_fill_hourly()

        result_combined = result.join(df_hourly, how='left')

        leaves_info_df = pd.DataFrame(leaves_info).sort_values(by='Leaf Number').reset_index(drop=True)

        removed_leaves_df = pd.DataFrame(removed_leaves_info)

        remaining_leaves_info = leaf_calculator.create_remaining_leaves_info(result_combined, leaves_info_df)

        transformed_data = leaf_calculator.transform_remaining_leaves_info(remaining_leaves_info)
        transformed_data, max_len = leaf_calculator.pad_transformed_data(transformed_data)
        columns = leaf_calculator.create_columns(max_len)
        final_leaves_info_df = pd.DataFrame(transformed_data, columns=columns)

        gross_photosynthesis_rates_df, total_gross_photosynthesis_summary_df =
calculate_photosynthesis_rates(result_combined, final_leaves_info_df)

        total_gross_photosynthesis_summary_df['Date'] = total_gross_photosynthesis_summary_df['Timestamp'].dt.date
        total_gross_photosynthesis_summary_df_day =
total_gross_photosynthesis_summary_df.groupby('Date')['total_gross_photosynthesis_amount/hr'].sum().reset_index()
        total_gross_photosynthesis_summary_df_day.rename(columns={'total_gross_photosynthesis_amount/hr':
'total_gross_photosynthesis_amount/day (umol/m^2/day)'}, inplace=True)

        # CH2O production
        total_gross_photosynthesis_summary_df_day['A_grossCH2O PRODUCTION (g/m^2/day)'] =
(total_gross_photosynthesis_summary_df_day['total_gross_photosynthesis_amount/day (umol/m^2/day)'] / 1000000) *
44.01 * 30 / 44

        # Removed_Leaf_Area
        total_gross_photosynthesis_summary_df_day['Removed_Leaf_Area']=
total_gross_photosynthesis_summary_df_day['Date'].apply(
            lambda date: removed_leaves_df[removed_leaves_df['Date'] == date]['Removed Leaf Area_m2'].sum() if date in
```

```
removed_leaves_df['Date'].values else 0
        )

        # Removed_Leaf_Area*(1/SLA) (g/m^2) calculate
        total_gross_photosynthesis_summary_df_day['Removed_Leaf_Area*(1/SLA)(g/m^2)']=
total_gross_photosynthesis_summary_df_day['Removed_Leaf_Area'] * (1 / SLA)

        # Rm_Vegetative and Rm_Fruit (CH2Og/g DM) calculate
        rm_df = calculate_rm(result_combined)
        total_gross_photosynthesis_summary_df_day = total_gross_photosynthesis_summary_df_day.merge(rm_df, on='Date',
how='left')

        # add Harvested Fruit DW (g/m^2) data

        total_gross_photosynthesis_summary_df_day = add_fruit_dw_column(total_gross_photosynthesis_summary_df_day,
fruit_dw_file_path, fruit_dw_sheet_name)

        # Partitioning to vegetative and fruit ratio calculation
        total_gross_photosynthesis_summary_df_day['Partitioning to vegetative ratio'] =
total_gross_photosynthesis_summary_df_day['Date'].apply(
            lambda date: partitioning_vegetative_before if date < pd.to_datetime(split_date).date() else 1 - (0.00786 *
daily_avg_temp.loc[date, 'temperature'] + 0.2886)
        )
        total_gross_photosynthesis_summary_df_day['Partitioning to fruit ratio'] =
total_gross_photosynthesis_summary_df_day['Date'].apply(
            lambda date: partitioning_fruit_before if date < pd.to_datetime(split_date).date() else (0.00786 *
daily_avg_temp.loc[date, 'temperature'] + 0.2886)
        )

        #setting initial vale of dry weight of whole plant
        total_gross_photosynthesis_summary_df_day.loc[0, 'Estimated Remaining Vegetative DW (g/m^2)'] =
initial_remaining_vegetative_dw
        total_gross_photosynthesis_summary_df_day.loc[0, 'Estimated Remaining fruit DW (g/m^2)'] = 0    # need to set initial
value

        # Calculate 'CH2O (g) for MAINTENIENCE RESPIRATION' and 'Total-Rm' on first day
        total_gross_photosynthesis_summary_df_day.loc[0, 'CH2O (g) for MAINTENIENCE RESPIRATION'] = (
            total_gross_photosynthesis_summary_df_day.loc[0, 'Estimated Remaining fruit DW (g/m^2)'] *
total_gross_photosynthesis_summary_df_day.loc[0, 'Rm_Fruit (CH2Og/g DM)'] +
            total_gross_photosynthesis_summary_df_day.loc[0, 'Estimated Remaining Vegetative DW (g/m^2)'] *
```

```
611 total_gross_photosynthesis_summary_df_day.loc[0, 'Rm_Vegetative (CH2Og/g DM)']
612         )
613         total_gross_photosynthesis_summary_df_day.loc[0, 'Total-Rm'] = (
614             total_gross_photosynthesis_summary_df_day.loc[0, 'A_gross CH2O PRODUCTION (g/m^2/day)'] -
615 total_gross_photosynthesis_summary_df_day.loc[0, 'CH2O (g) for MAINTENIENCE RESPIRATION']
616         )
617         total_gross_photosynthesis_summary_df_day.loc[0, 'Dry Matter Production (g/m^2/day)'] = (
618             total_gross_photosynthesis_summary_df_day.loc[0, 'Total-Rm'] / 1.45
619         )
620
621         total_gross_photosynthesis_summary_df_day.loc[0, 'Estimated Vegetative DW Production (g/m^2/d)'] = (
622             total_gross_photosynthesis_summary_df_day.loc[0, 'Dry Matter Production (g/m^2/day)'] *
623 total_gross_photosynthesis_summary_df_day.loc[0, 'Partitioning to vegetative ratio']
624         )
625         total_gross_photosynthesis_summary_df_day.loc[0, 'Estimated Fruit DW Production (g/m^2/d)'] = (
626             total_gross_photosynthesis_summary_df_day.loc[0, 'Dry Matter Production (g/m^2/day)'] *
627 total_gross_photosynthesis_summary_df_day.loc[0, 'Partitioning to fruit ratio']
628         )
629
630         total_gross_photosynthesis_summary_df_day.loc[0, 'Estimated Remaining Vegetative DW (g/m^2)'] = (
631             total_gross_photosynthesis_summary_df_day.loc[0, 'Estimated Vegetative DW Production (g/m^2/d)'] -
632 total_gross_photosynthesis_summary_df_day.loc[0, 'Removed_Leaf_Area*(1/SLA) (g/m^2)']
633         )
634         total_gross_photosynthesis_summary_df_day.loc[0, 'Estimated Remaining fruit DW (g/m^2)'] = (
635             total_gross_photosynthesis_summary_df_day.loc[0, 'Estimated Fruit DW Production (g/m^2/d)'] -
636 total_gross_photosynthesis_summary_df_day.loc[0, 'Harvested Fruit DW (g/m^2)']
637         )
638
639           # set to 0 if a negative value is encountered
640         total_gross_photosynthesis_summary_df_day.loc[0, 'Estimated Remaining Vegetative DW (g/m^2)'] = max(0,
641 total_gross_photosynthesis_summary_df_day.loc[0, 'Estimated Remaining Vegetative DW (g/m^2)'])
642         total_gross_photosynthesis_summary_df_day.loc[0, 'Estimated Remaining fruit DW (g/m^2)'] = max(0,
643 total_gross_photosynthesis_summary_df_day.loc[0, 'Estimated Remaining fruit DW (g/m^2)'])
644
645
646         for i in range(1, len(total_gross_photosynthesis_summary_df_day)):
647             prev_vegetative_dw = total_gross_photosynthesis_summary_df_day.loc[i - 1, 'Estimated Remaining Vegetative
648 DW (g/m^2)']
649             prev_fruit_dw = total_gross_photosynthesis_summary_df_day.loc[i - 1, 'Estimated Remaining fruit DW (g/m^2)']
```

```
650
651             total_gross_photosynthesis_summary_df_day.loc[i, 'CH2O (g) for MAINTENIENCE RESPIRATION'] = (
652                 prev_fruit_dw * total_gross_photosynthesis_summary_df_day.loc[i, 'Rm_Fruit (CH2Og/g DM)'] +
653                 prev_vegetative_dw * total_gross_photosynthesis_summary_df_day.loc[i, 'Rm_Vegetative (CH2Og/g DM)']
654             )
655
656             total_gross_photosynthesis_summary_df_day.loc[i, 'Total-Rm'] = (
657                 total_gross_photosynthesis_summary_df_day.loc[i, 'A_gross CH2O PRODUCTION (g/m^2/day)'] -
658                 total_gross_photosynthesis_summary_df_day.loc[i, 'CH2O (g) for MAINTENIENCE RESPIRATION']
659             )
660
661             total_gross_photosynthesis_summary_df_day.loc[i, 'Dry Matter Production (g/m^2/day)'] = (
662                 total_gross_photosynthesis_summary_df_day.loc[i, 'Total-Rm'] / 1.45
663             )
664
665             total_gross_photosynthesis_summary_df_day.loc[i, 'Estimated Vegetative DW Production (g/m^2/d)'] = (
666                 total_gross_photosynthesis_summary_df_day.loc[i, 'Dry Matter Production (g/m^2/day)'] *
667                 total_gross_photosynthesis_summary_df_day.loc[i, 'Partitioning to vegetative ratio']
668             )
669
670             total_gross_photosynthesis_summary_df_day.loc[i, 'Estimated Fruit DW Production (g/m^2/d)'] = (
671                 total_gross_photosynthesis_summary_df_day.loc[i, 'Dry Matter Production (g/m^2/day)'] *
672                 total_gross_photosynthesis_summary_df_day.loc[i, 'Partitioning to fruit ratio']
673             )
674
675             total_gross_photosynthesis_summary_df_day.loc[i, 'Estimated Remaining Vegetative DW (g/m^2)'] = (
676                 prev_vegetative_dw + total_gross_photosynthesis_summary_df_day.loc[i, 'Estimated Vegetative DW
677 Production (g/m^2/d)'] -
678                 total_gross_photosynthesis_summary_df_day.loc[i, 'Removed_Leaf_Area*(1/SLA) (g/m^2)']
679             )
680
681             total_gross_photosynthesis_summary_df_day.loc[i, 'Estimated Remaining fruit DW (g/m^2)'] = (
682                 prev_fruit_dw + total_gross_photosynthesis_summary_df_day.loc[i, 'Estimated Fruit DW Production
683 (g/m^2/d)'] -
684                 total_gross_photosynthesis_summary_df_day.loc[i, 'Harvested Fruit DW (g/m^2)']
685             )
686
```

```
            total_gross_photosynthesis_summary_df_day.loc[i, 'Estimated Remaining Vegetative DW (g/m^2)'] = max(0, total_gross_photosynthesis_summary_df_day.loc[i, 'Estimated Remaining Vegetative DW (g/m^2)'])
            total_gross_photosynthesis_summary_df_day.loc[i, 'Estimated Remaining fruit DW (g/m^2)'] = max(0, total_gross_photosynthesis_summary_df_day.loc[i, 'Estimated Remaining fruit DW (g/m^2)'])

            print(f"Row {i}:")
            print(total_gross_photosynthesis_summary_df_day.iloc[i])

        with pd.ExcelWriter(output_file_path) as writer:
            result_combined.reset_index().rename(columns={'index':'DateTime'}).to_excel(writer,sheet_name='Results', index=False)
            leaves_info_df.to_excel(writer, sheet_name='Leaf_Info', index=False)
            removed_leaves_df.to_excel(writer, sheet_name='Removed_Leaf_Info', index=False)
            final_leaves_info_df.to_excel(writer, sheet_name='Remaining_Leaves_Info', index=False)
            gross_photosynthesis_rates_df.to_excel(writer, sheet_name='Rank_Photosynthesis_Rates_GROSS', index=False)
            total_gross_photosynthesis_summary_df.to_excel(writer,sheet_name='Total_Photosynthesis_GROSS', index=False)
            total_gross_photosynthesis_summary_df_day.to_excel(writer,sheet_name='Daily_Photosynthesis_GROSS', index=False)

        print(f" result save in {output_file_path}")
        return total_gross_photosynthesis_summary_df_day
```

Simulation run code

```
file_path = 'file path with input environmental data’
sheet_name = 'Sheet name of input environmental data’'
start_date = '2021-02-23'
initial_nodes = 3
threshold_before = 26.3
threshold_after = 15.6
split_date = '2021-03-26'
leaf_removal_dates_file = 'file path with leaf removal date information’
leaf_removal_sheet_name = 'leaf removal sheet name'
output_file_path = ‘file path to save result’
SLA = 0.025
fruit_dw_file_path = ‘file path with daily harvested fruit weight information’
```

```
fruit_dw_sheet_name = '2'
partitioning_vegetative_before = 1.0
partitioning_fruit_before = 0.0
initial_remaining_vegetative_dw = 1.16
plant_density_per_m2 = 1.72

total_gross_photosynthesis_summary_df_day = simulate_photosynthesis(
    file_path, sheet_name, start_date, initial_nodes, threshold_before, threshold_after, split_date,
    leaf_removal_dates_file, leaf_removal_sheet_name, output_file_path, SLA,
    fruit_dw_file_path, fruit_dw_sheet_name, partitioning_vegetative_before, partitioning_fruit_before,
    initial_remaining_vegetative_dw, plant_density_per_m2)

```

Supplementary information 7

To calculate the curvature of the light response of J (θ) for each cropping type and leaf position, the respective light response curve data were used, and Eqn. S7-1, S7-2, S7-3, S7-4, and S7-5 (Ziegler-Jöns and Selinger 1987).

$$\theta J^2 - (J_m + I')J + J_m I' = 0 \quad \text{(S7-1)}$$

$$\mathrm{A} = \Pi \mathrm{J} - D^1 \quad \text{(S7-2)}$$

$$\Pi = (p^c - \Gamma^*)/(4.5p^c + 10.5\Gamma^*) \quad \text{(S7-3)}$$

$$\left(\frac{dJ}{dI}\right)_{I\to 0} = \frac{\varepsilon(1-f)}{2} = q \quad \text{(S7-4)}$$

$$I' = qI = \frac{I}{\Pi} \cdot \left(\frac{\partial A}{\partial I}\right)_{I\to 0} \quad \text{(S7-5)}$$

J_m is the maximum rate of whole electron transport, I′ is effective photosynthetic photon flux density (PPFD), A is net photosynthesis, Π is conversion factor for calculation of CO_2 uptake, D^1 is the rate of disseminative respiration occurring in the light, p^c is the intercellular CO_2 partial pressure, Γ^* is CO_2 compensation point in the absence of dissimilative respiration (μbar), ε is absorption coefficient, f is the loss factor for the use of white light instead of red, and q is effectivity factor for the use of light (electrons/quanta).

Supplementary information 8

To obtain the ratio of J_{max25}/V_{cmax25}, we used the A/C_i data measured 17 times in total on a PPFD 1500 (μmol m^{-2} s^{-1}) from December 3, 2021 to April 21, 2022 in Experiment 2, using the middle-position leaves of *'Hangangmatbaekdadagi'* as the material. The measurement method is the same as that in the Materials and Methods section in main text except for the set light intensity. Using the data, V_{cmax25} and J_{max25} of the FvCB model were estimated to be 70.85 and 122.25, respectively (R^2 = 0.95, RMSE = 3.07) (Fig. S8), resulting in a ratio of J_{max25}/V_{cmax25} of 1.7.

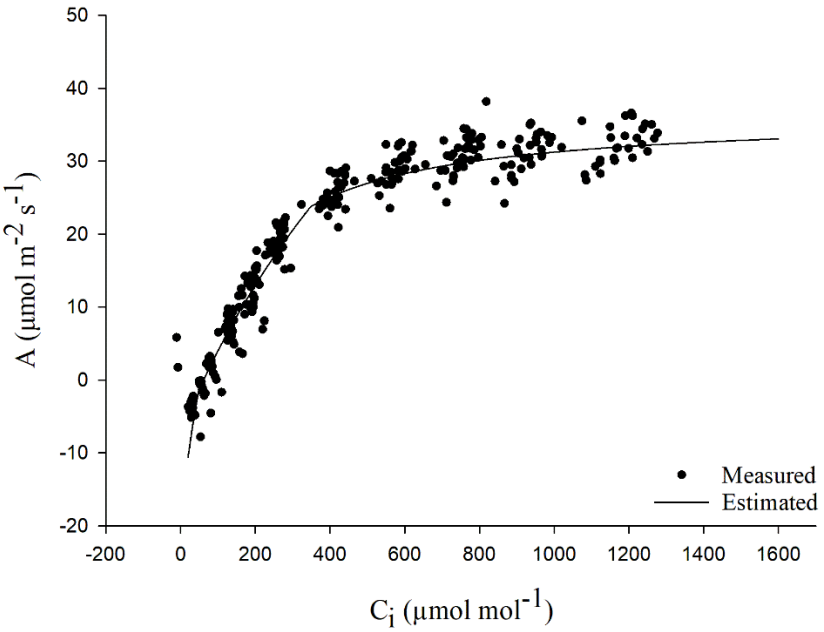

Figure S8. Results of FvCB model and measured data of middle leaf under forcing cropping type at PPFD 1500 (μmol m^{-2} s^{-1})

LITERATURE CITED

Marcelis LFM. **1994**. Effect of fruit growth, temperature and irradiance on biomass allocation to the vegetative parts of cucumber. *Netherlands Journal of Agricultural Science* **42**: 115–123.

Qian T, Elings A, Dieleman JA, Gort G, Marcelis LFM. **2012**. Estimation of photosynthesis parameters for a modified Farquhar–von Caemmerer–Berry model using simultaneous estimation method and nonlinear mixed effects model. *Environmental and Experimental Botany* **82**: 66–73.

Ziegler-Jöns A, Selinger H. **1987**. Calculation of leaf photosynthetic parameters from light-response curves for ecophysiological applications. *Planta* **171**: 412–415.

Qian T, Elings A, Dieleman JA, Gort G, Marcelis LFM. **2012**. Estimation of photosynthesis parameters for a modified Farquhar–von Caemmerer–Berry model using simultaneous estimation method and nonlinear mixed effects model. *Environmental and Experimental Botany* **82**: 66–73.

Ziegler-Jöns A, Selinger H. **1987**. Calculation of leaf photosynthetic parameters from light-response curves for ecophysiological applications. *Planta* **171**: 412–415.

Kalyanaraman A, Burnett M, Fern A, Khot L, Viers J. 2022. Special report: The AgAID AI institute for transforming workforce and decision support in agriculture. Computers and Electronics in Agriculture, 197.
