# Supplementary figures and images for: Simulation of dry matter partitioning in cucumber fruits: reflecting gas exchange characteristics based on leaf position and cropping type
Source: Hortic Res. 2025 May 7;12(8):uhaf124. doi: 10.1093/hr/uhaf124 (PMC12261107; doi:10.1093/hr/uhaf124)

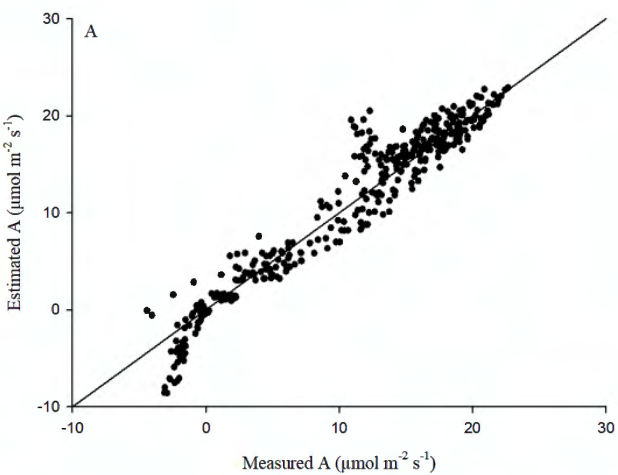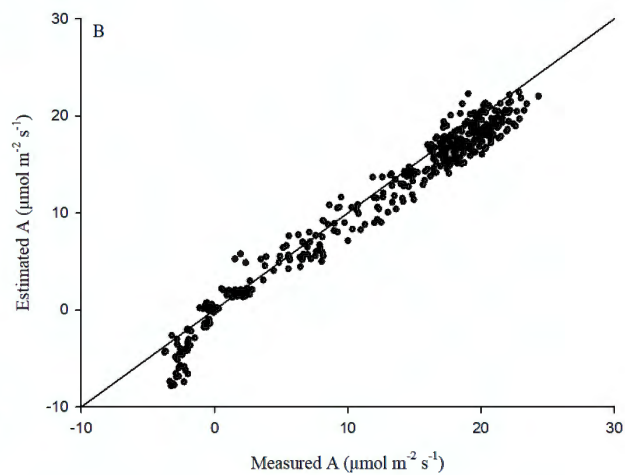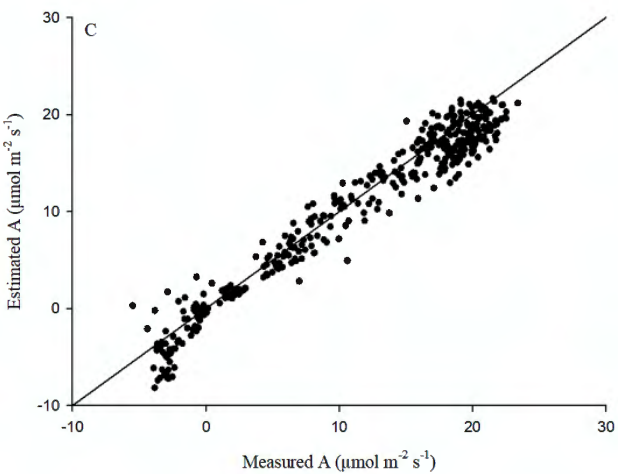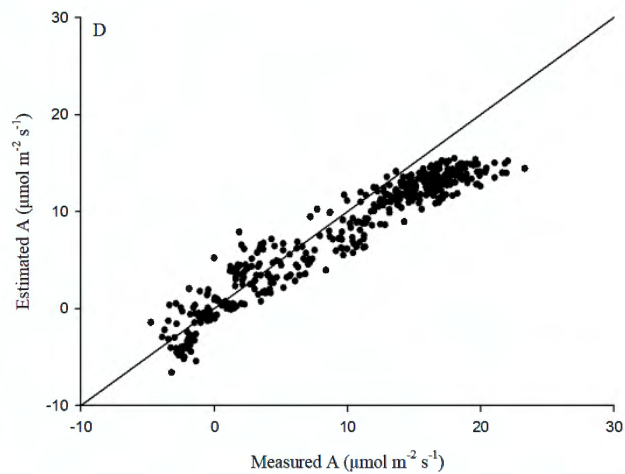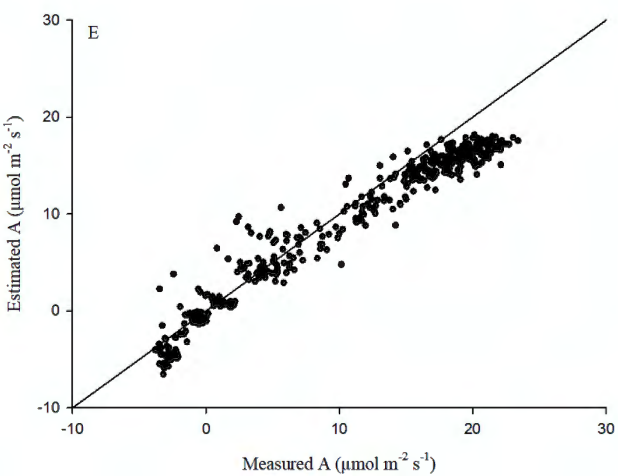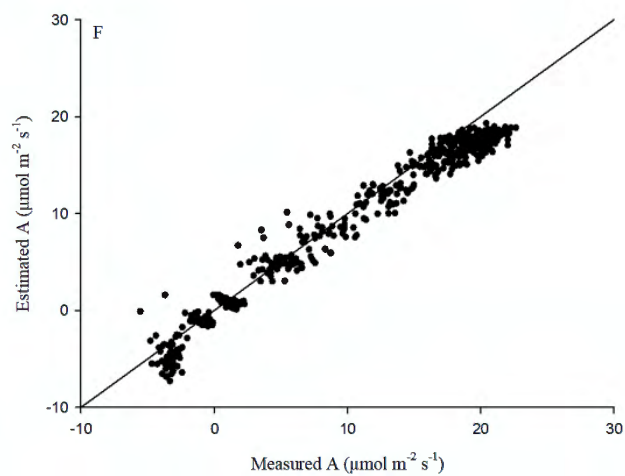

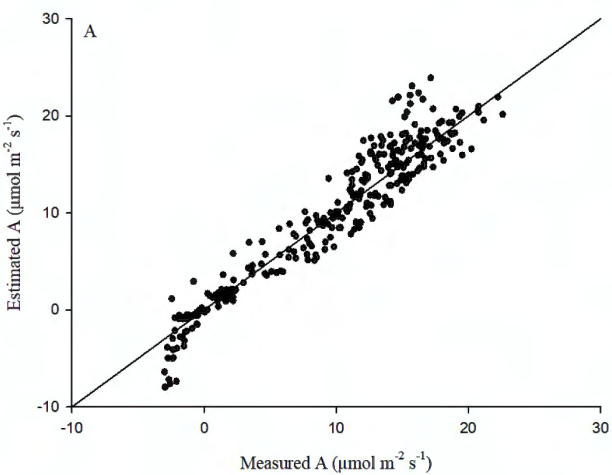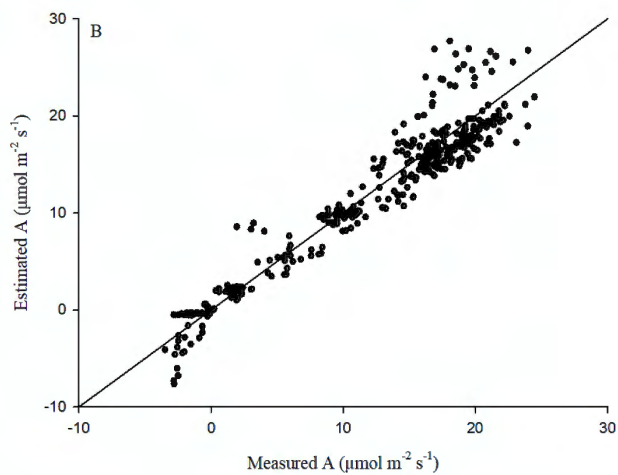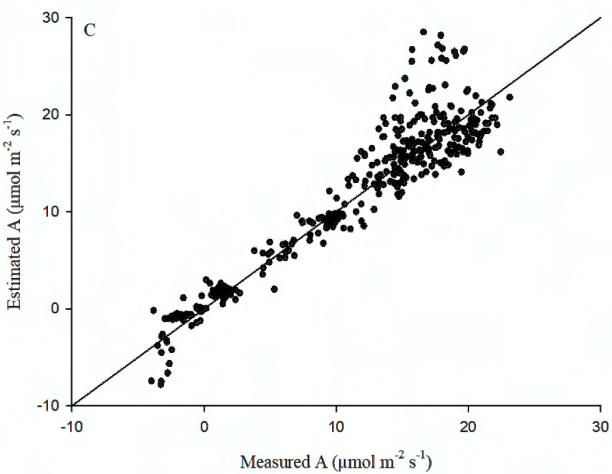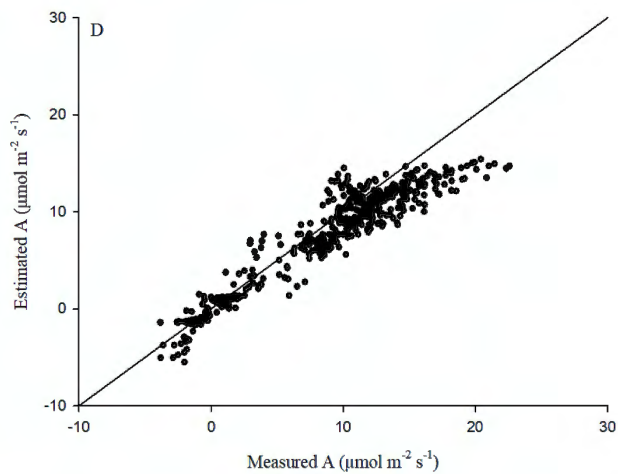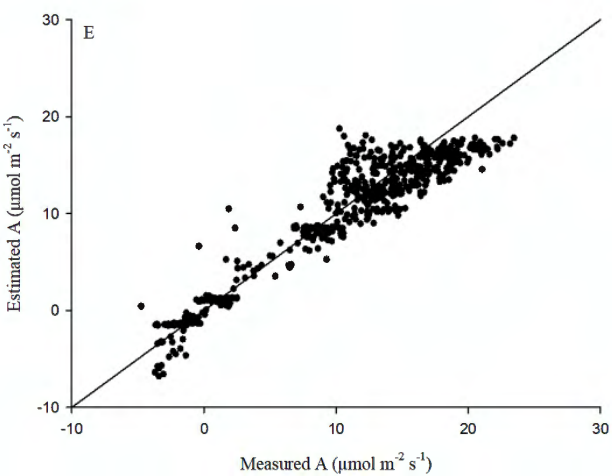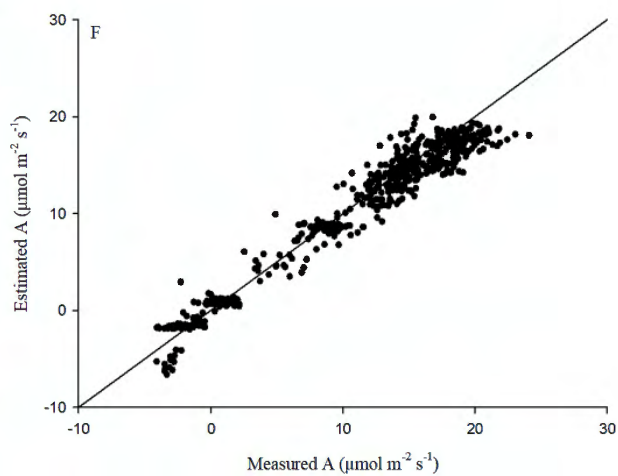

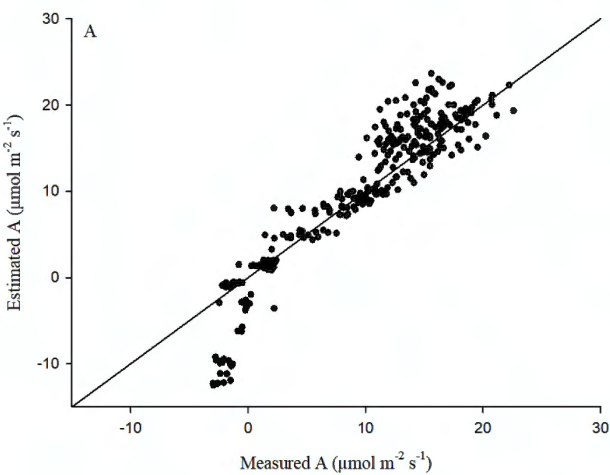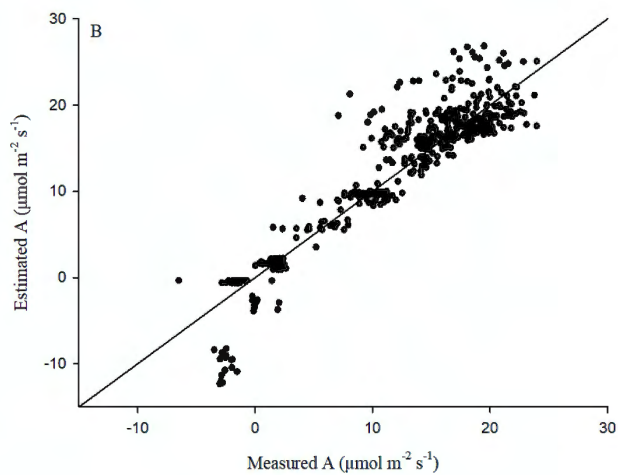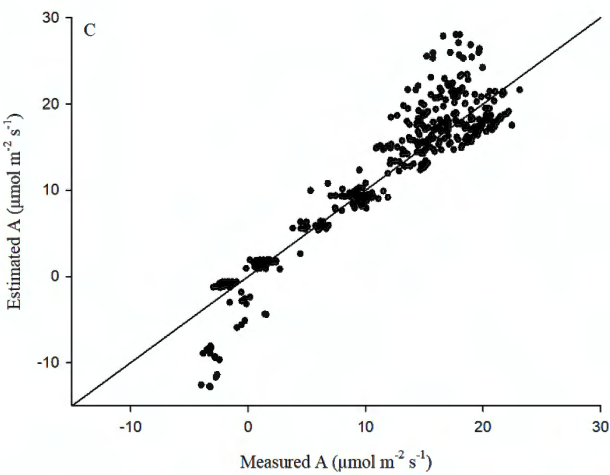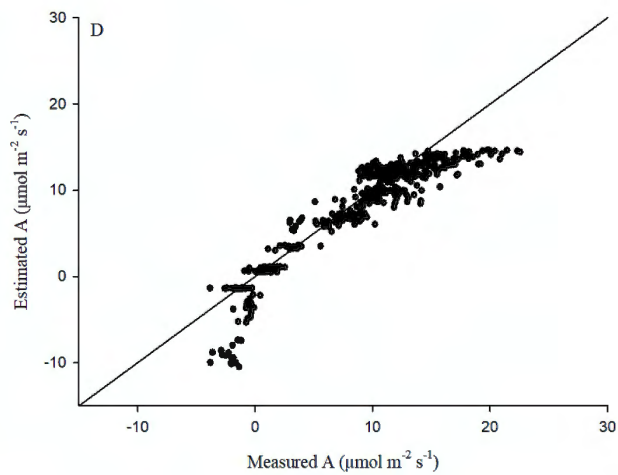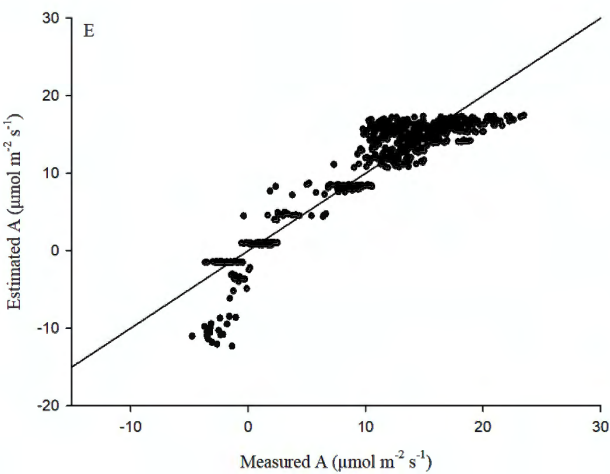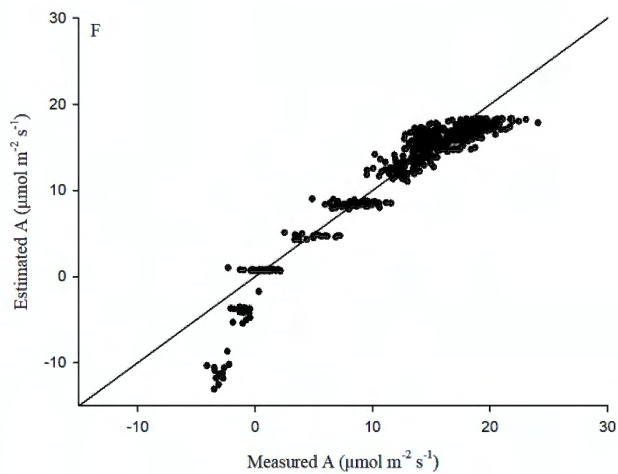

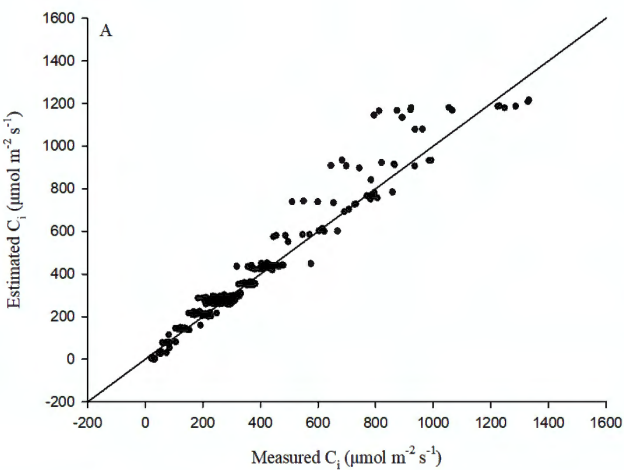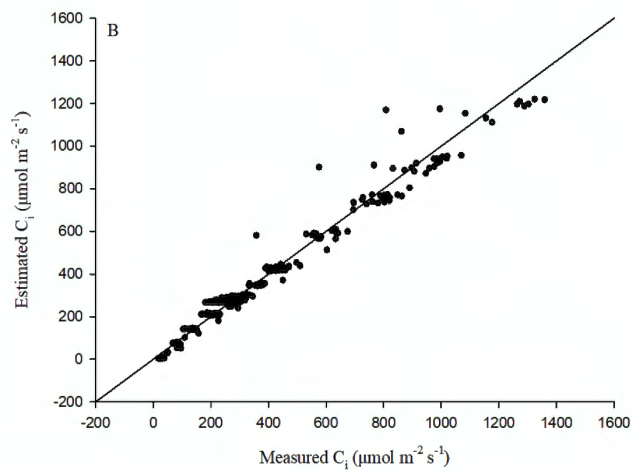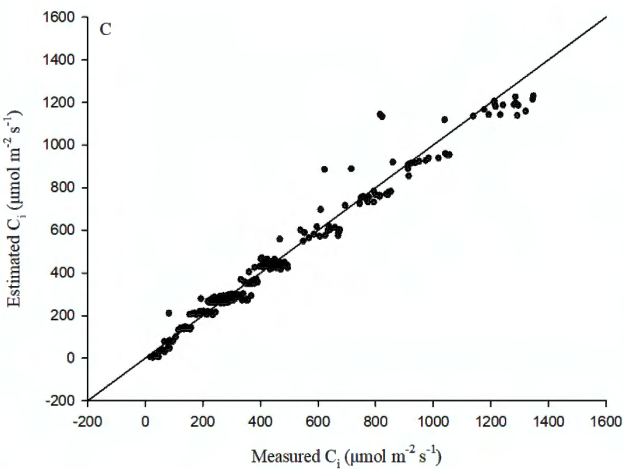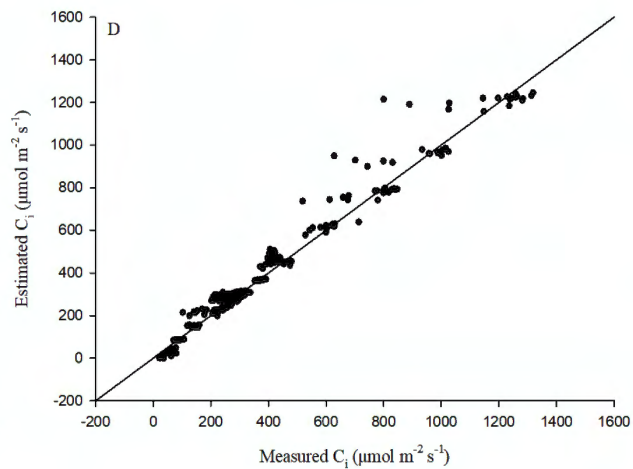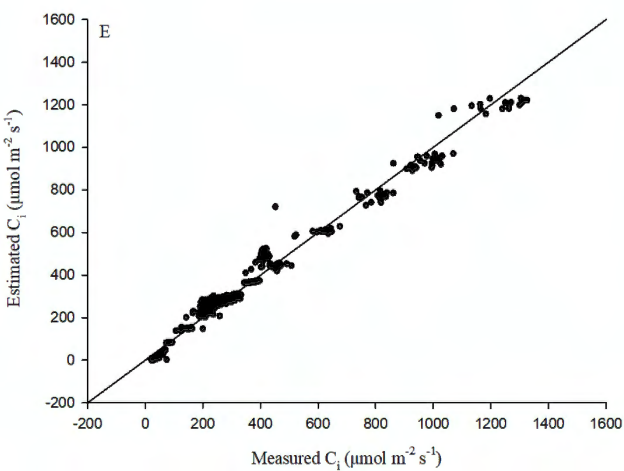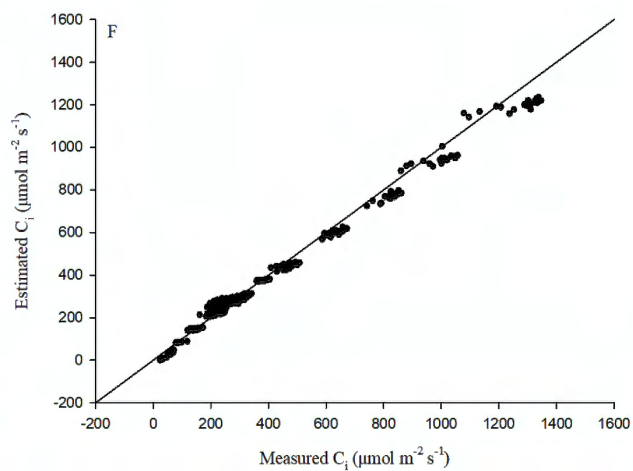

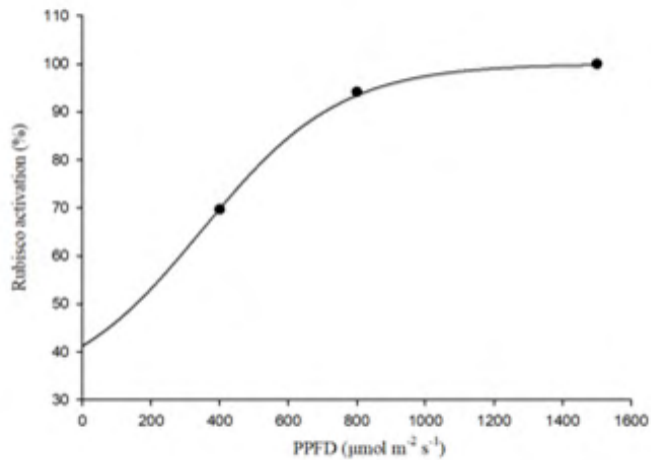

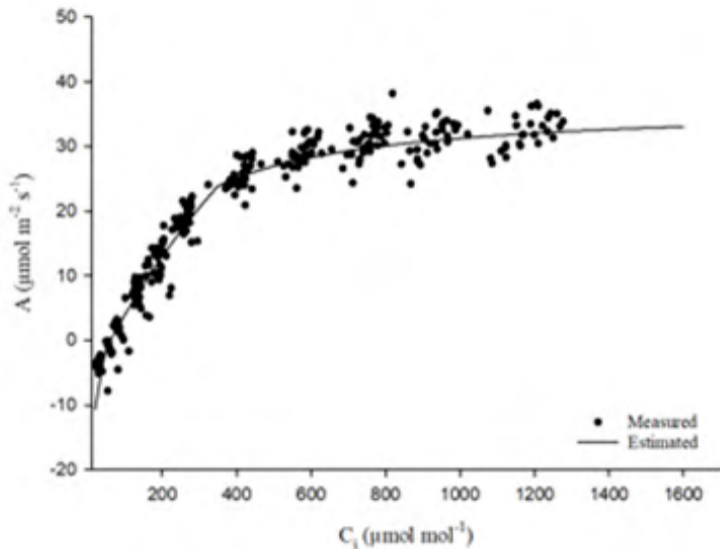

Supplement: Web_Material_uhaf124 [file web_material_uhaf124.zip › Supple figures_vf.pdf]
